# Exploiting Sodium Coordination in Alternating Monomer Sequences to Toughen Degradable Block Polyester Thermoplastic Elastomers

**DOI:** 10.1021/acs.macromol.2c00068

**Published:** 2022-03-03

**Authors:** Georgina L. Gregory, Charlotte K. Williams

**Affiliations:** Chemistry Research Laboratory, Department of Chemistry, University of Oxford, 12 Mansfield Road, Oxford OX1 3TA, U.K.

## Abstract

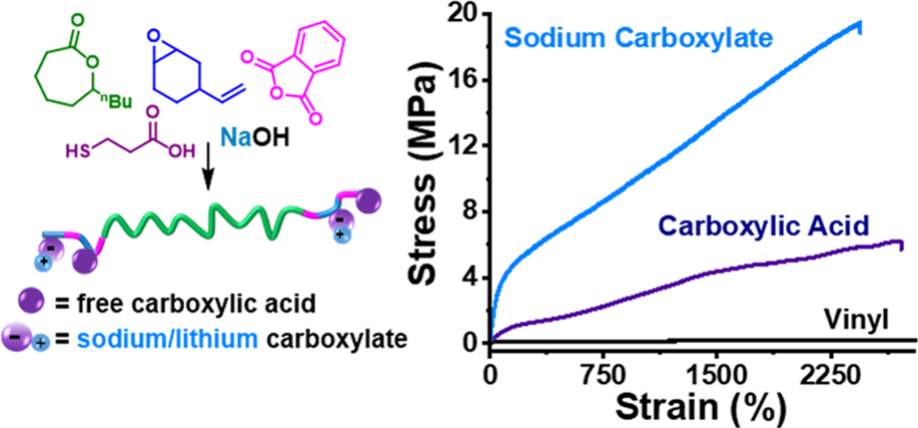

Thermoplastic elastomers (TPEs) that are closed-loop recyclable are needed in a circular material economy, but many current materials degrade during recycling, and almost all are pervasive hydrocarbons. Here, well-controlled block polyester TPEs featuring regularly placed sodium/lithium carboxylate side chains are described. They show significantly higher tensile strengths than unfunctionalized analogues, with high elasticity and elastic recovery. The materials are prepared using controlled polymerizations, exploiting a single catalyst that switches between different polymerization cycles. ABA block polyesters of high molar mass (60–100 kg mol^–1^; 21 wt % A-block) are constructed using the ring-opening polymerization of ε-decalactone (derived from castor oil; B-block), followed by the alternating ring-opening copolymerization of phthalic anhydride with 4-vinyl-cyclohexene oxide (A-blocks). The polyesters undergo efficient functionalization to install regularly placed carboxylic acids onto the A blocks. Reacting the polymers with sodium or lithium hydroxide controls the extent of ionization (0–100%); ionized polymers show a higher tensile strength (20 MPa), elasticity (>2000%), and elastic recovery (>80%). In one case, sodium functionalization results in 35× higher stress at break than the carboxylic acid polymer; in all cases, changing the quantity of sodium tunes the properties. A leading sample, **2**-COONa75 (*M*_n_ 100 kg mol^–1^, 75% sodium), shows a wide operating temperature range (−52 to 129 °C) and is recycled (×3) by hot-pressing at 200 °C, without the loss of mechanical properties. Both the efficient synthesis of ABA block polymers and precision ionization in perfectly alternating monomer sequences are concepts that can be generalized to many other monomers, functional groups, and metals. These materials are partly bioderived and have degradable ester backbone chemistries, deliver useful properties, and allow for thermal reprocessing; these features are attractive as future sustainable TPEs.

## Introduction

Pervasive, petrochemical-derived plastics have negative environmental impacts because their structures and chemistries were optimized for applications but not end of life. Their redesign, underpinned by sustainability considerations throughout their lifecycles, should seek to maximize biobased/low-greenhouse-gas-emission raw materials. New plastic properties and processing should match the existing products and infrastructure, facilitate efficient closed-loop recycling, and deliver chemistries that can completely deconstruct to small molecules/monomers (with low energy input).^1−5^ One important class of polymers are thermoplastic elastomers (TPEs) or synthetic rubbers. These products combine thermal processability with high elasticity and are important alternatives to thermosets or vulcanized natural rubber, neither of which can undergo closed-loop recycling. They are widely used across construction, packaging, automotive, household goods, medical, and electronic sectors. Uses span sealants, gaskets, plugs, tubing, cables, heat-shrink films, tires, shoe soles, sports equipment, vehicle bumpers and interiors, medical tubing, catheters, and implants, to name a few.^6^ Currently, the most commercial TPEs are petrochemicals and are prepared using uncontrolled polymerizations that necessitate careful regulation of conditions to yield the desired phase-separated nanostructures.^7^ Developing controlled polymerizations is critical to improve the process efficiency;^4^ delivery of well-controlled polymer structures is essential for optimizing macroscopic properties.^8^ A long-standing challenge is to develop methods to increase TPE tensile strength and toughness without simultaneously reducing the elasticity and elastic recovery.^9−11^

ABA-type block polymers are a significant subclass of TPEs since they have well-controlled structures. The *B* polymer is usually amorphous and has a low glass transition temperature (*T*_g_) (“soft” block), while the *A* polymers may be semicrystalline or amorphous with higher melting/glass transition temperatures (“hard” block). Phase separation of the blocks into hard (A-block) and soft (B-block) domains is critical, and the structures should comprise a soft polymer matrix, imparting elasticity, with hard domains providing physical crosslinks conferring mechanical strength.^6^ One issue is that the tensile strength and extensibility tend to be inversely related. For example, increasing the A-block content results in stiffer but less stretchable materials.

Dynamic covalent bonds are useful in polymer chemistry to install impermanent and switchable crosslinks; successful examples include hydrogen-bonding, metal–ligand coordination, or ionic interactions.^12−20^ The concept remains underexplored in TPE chemistry, although hydrocarbon polymers bearing ∼1–15 mol % ions, known as ionomers, have long been known to show useful properties.^21^ For example, a poly(ethylene-*co*-methacrylic acid)-based TPE, developed by DuPont under the name Surlyn, shows impressive tensile strengths (22 MPa) when sodium cations partially neutralize the carboxylic acid groups.^22^ Partially ionized “soft” blocks in the poly(styrene-*b*-isoprene-*b*-styrene) (SIS) Kraton family of commercial TPEs show an excellent toughness of 480 MJ m^–3^.^23^ The limitation of these commercial ionomers is that both the polymerization methods and ionic group functionalization lack control; this means that material property optimization is somewhat empirical. Important questions remain regarding how the ionizable group, the extent of ionization, or choice of counterions influence the thermomechanical properties.^24−27^

We hypothesized that by installing regularly placed dynamic ionic interactions to a precision block polyester backbone, it should be possible to deliver fully recyclable and high-performance TPEs. Polyesters could also be useful since the ester linkages are well known to fully degrade under acidic, basic, or enzymatic conditions, thereby providing a future route for chemical recycling and/or biodegradation.^28−30^ Hillmyer and co-workers have pioneered block polyester TPEs using controlled ring-opening polymerization (ROP) of bioderived cyclic esters to make the ABA polymers. Soft-block polyesters (−60 < *T*_g_ < −25 °C) were prepared from menthide (mint),^31,32^ ε-decalactone (castor oil),^33^ 6-methyl-ε-caprolactone,^34^ β-methyl-δ-valerolactone (glucose), ε-caprolactone (petrochemical but biodegradable),^35^ and γ-methyl-ε-caprolactone (cresols from lignin).^36,37^ In all cases, the hard-block was polylactide, PL(L)A (corn starch, *T*_g_ = 60 °C, *T*_m_ = 130–160 °C), and stereocomplex formation [between the chains of poly(l-lactide) (PLLA) and poly(D-lactide) (PDLA)] increased the Young moduli and tensile strength by 2–3-fold.^38,39^ Although further improvements could be achieved using substituted lactones, these are not yet commercially available and, in some instances, suffer from low polymerization enthalpy.^40^

Recently, we reported degradable, semiaromatic polyesters prepared by ring-opening copolymerization (ROCOP) of phthalic anhydride (PA) and cyclohexene oxide (CHO).^41^ When combined with a poly(ε-decalactone) (PDL) soft block, polyester TPEs showed elastomeric properties matching those at the lower end of commercial styrenic copolymers.^42^ Replacing CHO with vinyl-cyclohexene oxide (vCHO) might allow for functionalization and ionization at the vinyl sites, improving the mechanical properties. vCHO is a commercial reagent with a precedent for efficient alternating copolymerization with anhydrides and postpolymerization functionalization to install carboxylic acids.^43−47^ Ion selection was constrained to inexpensive, earth-abundant, and light sodium or lithium.

## Results and Discussion

### Synthesis

**Figure 1:**
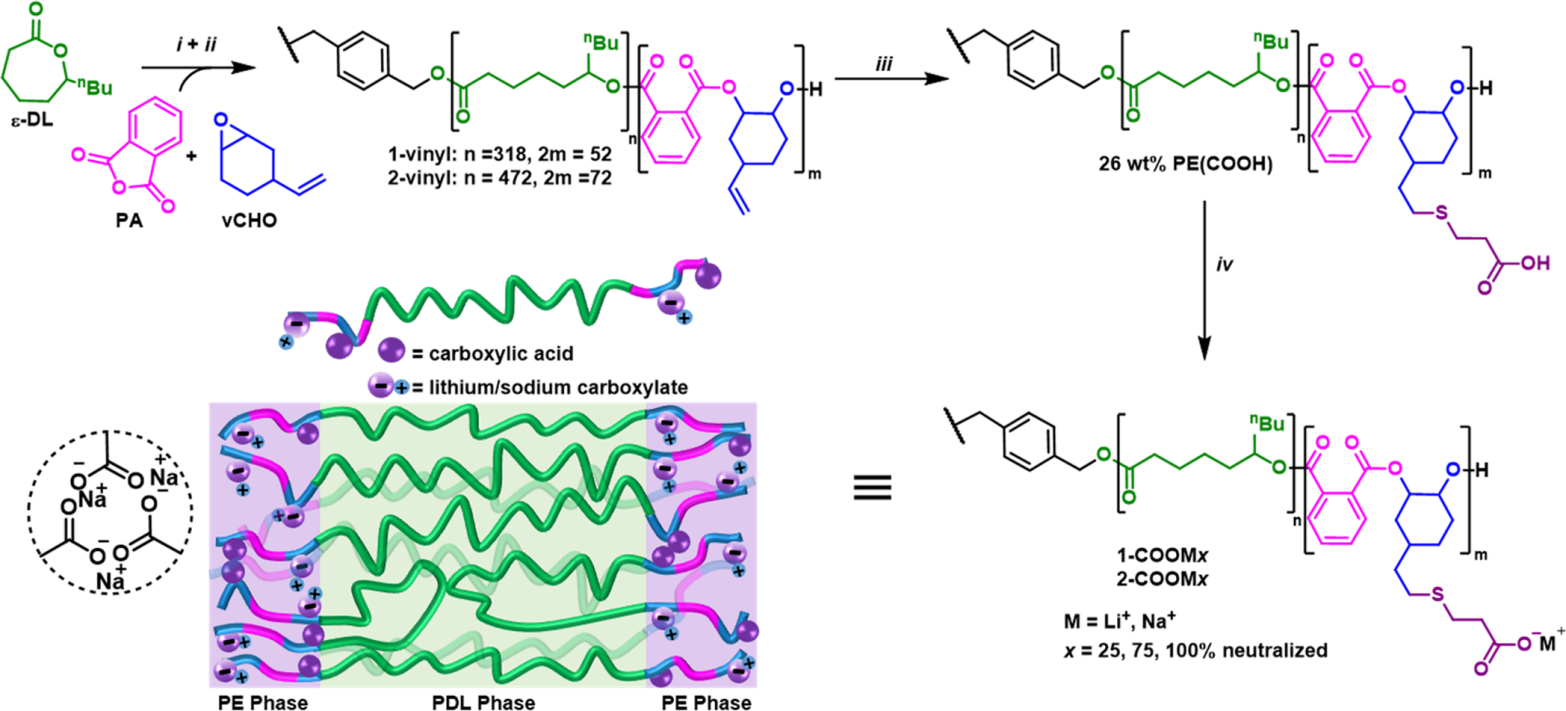
Synthesis of polyester ionomers. (i) DL ROP at 80 °C catalyzed by LZnMg(C_6_F_5_)_2_ (see the Supporting Information for the structure) with a 1,4-benzenedimethanol (BDM) bifunctional initiator, where [Cat]_0_/[BDM]_0_/[DL]_0_ = 1:4:2000 (**2**-vinyl) and [DL]_0_ = 2.0 M in toluene (see Table S1 for monomer conversions and reaction times). (ii) Addition of PA (400 equiv) and excess vCHO (1200 equiv). ROCOP conducted at 100 °C for 24 h. For PE(v)–PDL–PE(v) (referred to as **1**-vinyl), *M*_n,total by SEC_ = 60 kg mol^–1^ (*Đ* 1.04) with 21 wt % PE(v) by NMR spectroscopy. For **2**-vinyl, *M*_n,total by SEC_ = 100 kg mol^–1^ (*Đ* 1.05), 20 wt % PE(v). (iii) UV-mediated thiol–ene reaction (0.5 h) with 3-mercaptopropionic acid (MPA) and dimethoxy-2-phenylacetophenone photoinitiator. PE(COOH)–PDL–PE(COOH) (referred to as **1**-or **2**-COOH) corresponds to 25/26 wt % PE(COOH). (iv) Full or partial neutralization of the carboxylic acid with LiOH or NaOH.

ABA-triblock polyesters were efficiently synthesized using a one-pot procedure operating with a single catalyst (Figure 1). This type of switchable polymerization allows monomer mixtures to be selectively enchained using the same catalyst to make specific block polymer sequences.^48^ In these reactions, adding anhydride to cyclic ester ROPs results in rapid anhydride insertion into the metal-alkoxide-propagating species, effectively switching the polymerization mechanism *in situ* and obviating intermediary isolation/purification.^49^ Here, the switchable catalytic selectivity was exploited to prepare ABA (hard–soft–hard) block polyesters. The catalyst comprised a high-activity heterodinuclear Zn(II)Mg(II) complex featuring two organometallic ligands, that is, [LZnMg(C_6_F_5_)_2_]. It was applied with 1,4-benzene dimethanol (BDM) and formed the active initiator, a metal alkoxide, *in situ* (Scheme S1 for the catalyst structure). This catalyst shows both high activity and tolerance, meaning that it is particularly well suited for producing higher-molar-mass polymers and operates without any cocatalyst, giving the highest control over end-group chemistry.^42,50^ End-group control yielding only hydroxyl telechelic chains delivers the desired ABA structure (where the cocatalysts would lead to contamination by lower block sequences, *e.g.*, AB polymers). Both polymerizations are very well controlled, meaning that each polymer block achieves predictable degrees of polymerization. Polymerizations were conducted by adding ε-decalactone (DL), dissolved in toluene, to the Zn(II)Mg(II) catalyst and BDM. The ROP of DL proceeded to form the soft-block PDL (B-block), as judged by aliquot analysis (TOF = 1000 h^–1^). PA was then added to switch the polymerization mechanism into the vCHO/PA ROCOP cycle and install the semiaromatic polyester end blocks (A-blocks). Monomer conversion was monitored by aliquot analysis (^1^H NMR spectroscopy) throughout the reaction. Once complete, the reaction was quenched and the final polymer isolated by precipitation from methanol.

The successful formation of the ABA triblock polyester was confirmed using a range of techniques. Polymer end groups were titrated using ^31^P{^1^H} NMR spectroscopy, and the final block polyesters showed only A-blocks, that is, poly(vinyl-cyclohexene oxide-*alt*-phthalic anhydride), henceforth referred to as PE(v) (Figure S1). Further support for selective ABA polyester formation came from observing a single diffusion coefficient for the polymer signals by diffusion ordered NMR spectroscopy (Figure S2). Size-exclusion chromatography (SEC) analysis of the aliquots showed an increase in polymer molar mass as the polymerization progressed and the continual evolution of narrow dispersity and monomodal distributions (Figure S3). The hard block, PE(v), content was targeted at 21 wt % as the analogous cyclohexene oxide-containing copolymer was a TPE at this composition.^42^ The experimental polymer composition closely matched the starting monomer ratios, consistent with well-controlled polymerizations, as determined by relative integration of proton environments specific to PDL (4.85 ppm) and vCHO (5.83 ppm) in the ^1^H NMR spectrum of the purified polymer. Even after repeated precipitations, the polymer composition remained constant, consistent with selective block polymer formation (Figure S4). By varying the catalyst:monomer ratios, two different triblock polyesters were produced, showing an overall *M*_n_ value of 60 (**1**-vinyl) or 100 kg mol^–1^ (**2**-vinyl) (Table S1).

The radical-mediated thiol–ene reaction with MPA installed carboxylic acid groups onto every vinyl-CHO unit in the hard PE(v) segment. After precipitation from methanol, the acid-functionalized polymer was characterized using ^1^H NMR spectroscopy (Figure S5). The data confirm the complete loss of the vinyl alkene resonances (5.83 ppm) and the appearance of new resonances assigned to the alkylene bridge moieties (2.56, 2.57 ppm). A new signal at 177.8 ppm, in the ^13^C{^1^H} NMR spectrum, is characteristic of the carboxylic acid carbonyl groups. No additional signals were observed for the block carbonyl groups, indicating that no polyester backbone transesterification occurred under these conditions (Figure S6). The SEC traces show slightly broadened molar mass distributions for the carboxylic acid versus vinyl-substituted polymers. However, importantly, the distributions remained monomodal with no higher *M*_n_ tailing, indicating little/no vinyl crosslinking as side reactions (Figure S7). The distribution broadening is tentatively attributed to changes to the polymer hydrodynamic radii on introducing the hydrophilic carboxylic acid.

**Figure 2:**
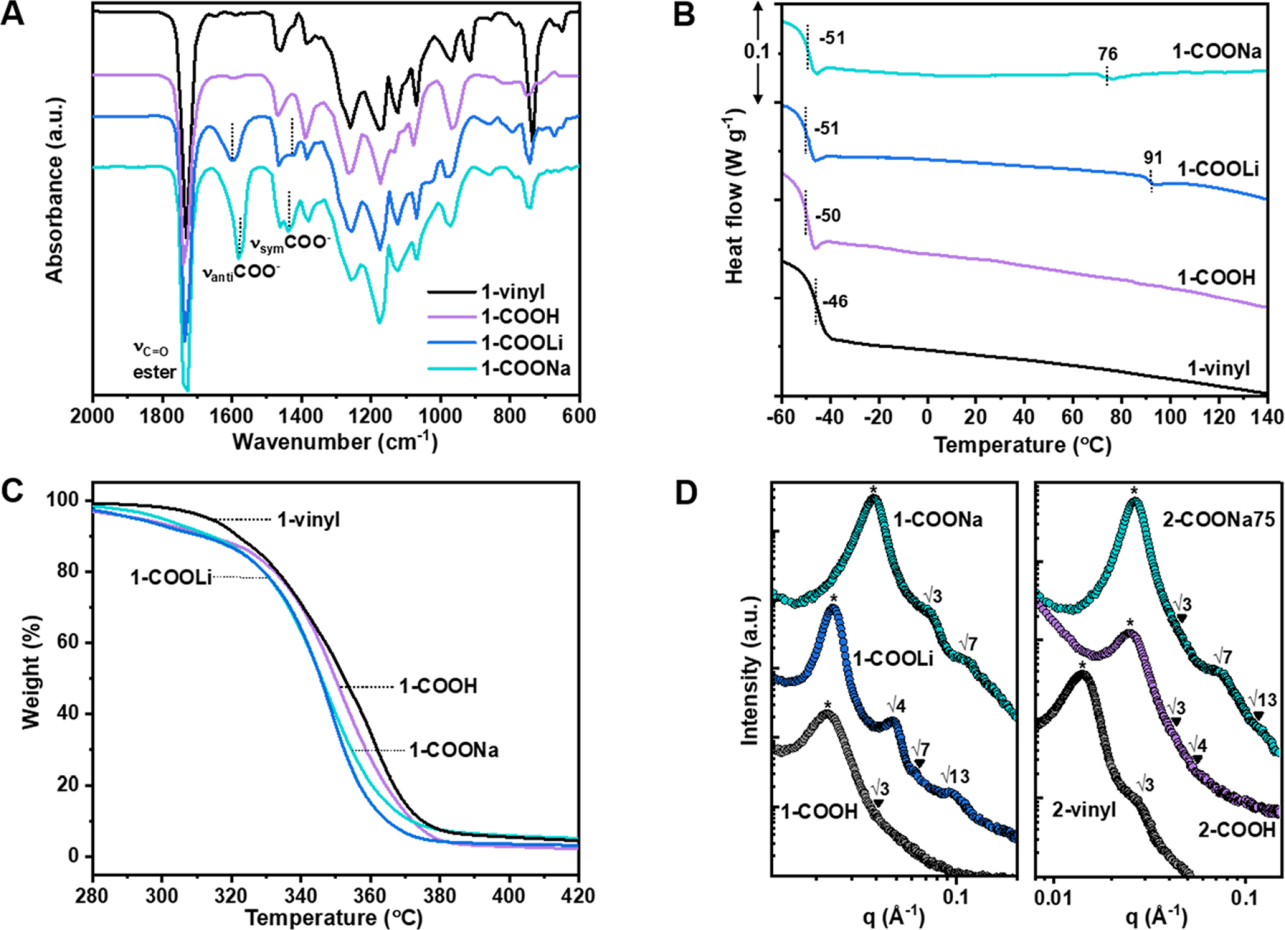
Characterization of block polyester ionomers. (A) FTIR spectra of polymer films comparing the unmodified (**1**-vinyl), carboxylic acid-functionalized (**1**-COOH), and fully lithium/sodium neutralized carboxylates (**1**-COOLi and **1**-COONa). (B) Differential scanning calorimetry (DSC) thermograms (measured between −80 and 200 °C at 10 °C min^–1^). (C) Thermogravimetric analysis (TGA) curves (10 °C min^–1^ heating rate). (D) Room-temperature small-angle X-ray scattering (SAXS) profiles with labeled principal scattering peaks (*) and higher-order peaks (*q*/q*) (Table S3).

The pendant carboxylic acid substituents were neutralized to pH 7 by reaction with lithium or sodium hydroxide; the reaction progress was monitored using a pH meter. The neutralized solution was poured into a Teflon mold and dried overnight at room temperature, before being dried in a vacuum oven at 80 °C, until a constant mass was achieved (72 h). Fourier-transform infrared (FTIR) spectroscopic analysis of the resulting polymer films showed new absorbances, characteristic of the carboxylate salt antisymmetric and symmetric stretches. These were observed at 1595 and 1422 cm^–1^ for the lithium carboxylate and 1580 and 1433 cm^–1^ for the sodium carboxylate, respectively (Figure 2A).^20,23^ The effect of the extent of sodium neutralization of the carboxylic acids was also investigated using **1**-COOH. Neutralization extents from 25, 50, and 75 mol % (*vs* COOH) were achieved by adding substoichiometric quantities of sodium hydroxide versus the starting carboxylic acid concentration and were verified by the relative band integrals of sodium carboxylate at 1580 cm^–1^ and polyester carbonyl signal at 1730 cm^–1^ in the FTIR spectra (Figure S8). ICP analysis was also used to verify the Na/Li content (Table S2). The ionomers were soluble in a mixture of THF and water. Lithium or sodium ions were also confirmed in all samples using either ^7^Li or ^23^Na NMR spectroscopy of the polymer films dissolved in 1:1 THF/D_2_O (Figure S9). SEC data showed that the neutralization process did not affect the backbone chemistry; for example, the molar mass distribution of **1**-COONa25 remained monomodal with comparable *M*_n_ to **1**-COOH (Figure S10).

### Thermal Properties

The key parameters for elastomeric applications are the polymers’ thermal transitions and potential for block phase separation. As expected, the polymers all showed completely amorphous structures since the polymerization is regiorandom and atactic, and both constituent polymer blocks are amorphous. DSC of the vinyl-functionalized triblock polymers shows a glass transition temperature (*T*_g_) at −46 °C, close to the value for pure PDL (−51 °C)^51^ and consistent with block phase separation (Figure 2B). There was no discernible upper *T*_g_ for the semiaromatic PE(v), but such phenomena are commonly observed for these amorphous “hard” blocks.^42^ Postpolymerization functionalization of the latter domain with the hydrophilic carboxylic acid lowers the PDL domain glass transition temperature slightly to −51 °C, suggesting marginally enhanced phase separation. The neutralized metal carboxylate polymers show both the lower *T*_g_ (−51 °C) and the upper *T*_g_ at 91 °C (Li) or 76 °C (Na), respectively. The higher glass transition temperature for the lithium carboxylate is consistent with smaller cations forming stronger ionic associations.^52^ Partial neutralization of the carboxylic acid moieties results in materials showing progressively increasing upper *T*_g_ values with neutralization extent (Figure S11). For the higher *M*_n_ polymer series (**2**-vinyl, **2**-COOH, and **2**-COONa75), two *T*_g_ values are observed in all cases and for **2**-COONa75 at −55 and 101 °C (Table 1 and Figure S11). All polymers show high thermal stability, with the onset of thermal degradation at temperatures >300 °C (Figures 2C and S12).

**Table 1:** Summary of the Key TPE Data^a^

sample^b^	σ_b_ (MPa)	ε_b_ (%)	*E*_y_ (MPa)	*U*_T_ (MJ m^–3^)	*T*_g1_, *T*_g2_ (°C)^c^	*T*_d,5%_ (°C)^d^
**1**-COOH	0.52 ± 0.03	2214 ± 20	1.1 ± 0.2	10.3 ± 0.6	–50, n.o	304
**1**-COONa75	17 ± 0.2	1919 ± 47	8.1 ± 1.5	166 ± 2	–51, 76	315
**2**-vinyl	1.02 ± 0.01	2785 ± 56	0.15 ± 0.01	∼2.1	–50, 140	328
**2**-COOH	5.8 ± 0.3	2747 ± 42	1.52 ± 0.02	97 ± 3	–50, 113 [−44, 120*]	323
**2**-COONa75	19.5 ± 0.2	2420 ± 28	8.8 ± 1.0	283 ± 8	–55, 101 [−52, 127*]	320
PE–PDL–PE (90, 23)^42^	6.5 ± 0.2	1097 ± 37	5.0 ± 0.7	40 ± 3	–51, 136*	307
PLLA–PDL–PLLA (191, 25)^54^	13.6 ± 0.5	1212 ± 25	2.9 ± 0.3	82 ± 5	–49, 60 (*T*_m_ 161)	225
PLLA/PDLA–PM–PLLA/PDLA^38^	21.8 ± 0.8	990 ± 62	29.2 ± 2.9	∼108	–21, (*T*_m_ 211)	
PLLA–PyMCL–PLLA (73, 28)^36^	35 ± 3	895 ± 20	13 ± 10	∼157	–59, 52 (*T*_m_ 162)	
PLLA–PCVL–PLLA^55^	46.3 ± 1.4	2100 ± 65	2.4	445 ± 12	(*T*_m_ 29, 160)	

a Tensile mechanical data of polymer films. *E*_y_ = Young’s modulus; ε_b_ = elongation at break; σ_b_ = tensile strength; *U*_T_ = tensile toughness (area under the stress–strain curve). ± is the standard deviation from at least three measurements.

b **1**- and **2**- refer to triblock polymers with *M*_n,SEC_ 60 or 100 kg mol^–1^, respectively; carboxylic acid-functionalized (**1/2**-COOH) or 75% neutralized with Na (**1/2**-COONa75). PE = poly(cyclohexene oxide-*alt*-phthalate) (*i.e.*, PA/CHO ROCOP); 90 or 105, the overall triblock *M*_n,SEC_ (kg mol^–1^), 18–23 wt % PE. PLLA = poly(l-lactide). PM = poly(menthide). PγMCL = poly(γ-methyl-ε caprolactone). PCVL = poly(ε-caprolactone-*co*-δ-valerolactone).

c Glass transition from DSC (10 °C min^–1^ heating rate, second heating curve). * *T*_g_ measured by DMTA from the peak in tan δ.

d Thermal degradation behavior measured by TGA; the temperature at which 5% mass is lost.

### Small-Angle X-ray Scattering

The phase morphology of the block polymer films was probed by SAXS conducted at room temperature (Figure 2D). The films were prepared by casting from THF or THF/water and drying in a vacuum oven (80 °C) for 72 h. From the principal scattering peak (q*), the calculated domain size (*d* = 2π/q*) was found to decrease from 28 nm for **1**-COOH to 26 nm on neutralization with LiOH and substantially for **1**-COONa (16 nm). Sharper, more intense q* and higher-order peaks are observed for the ionomers and their positions (*q*/q* = √3, √4, √7, √13) are evidence for hexagonally packed cylindrical morphologies (Table S3). Surprisingly, carboxylation of **2**-vinyl leads to a shift in q* to higher values (smaller domains), and a similar morphology to **1**-COONa is observed for **2**-COONa75 but with a larger domain spacing (24 nm).

### Mechanical Properties

**Figure 3:**
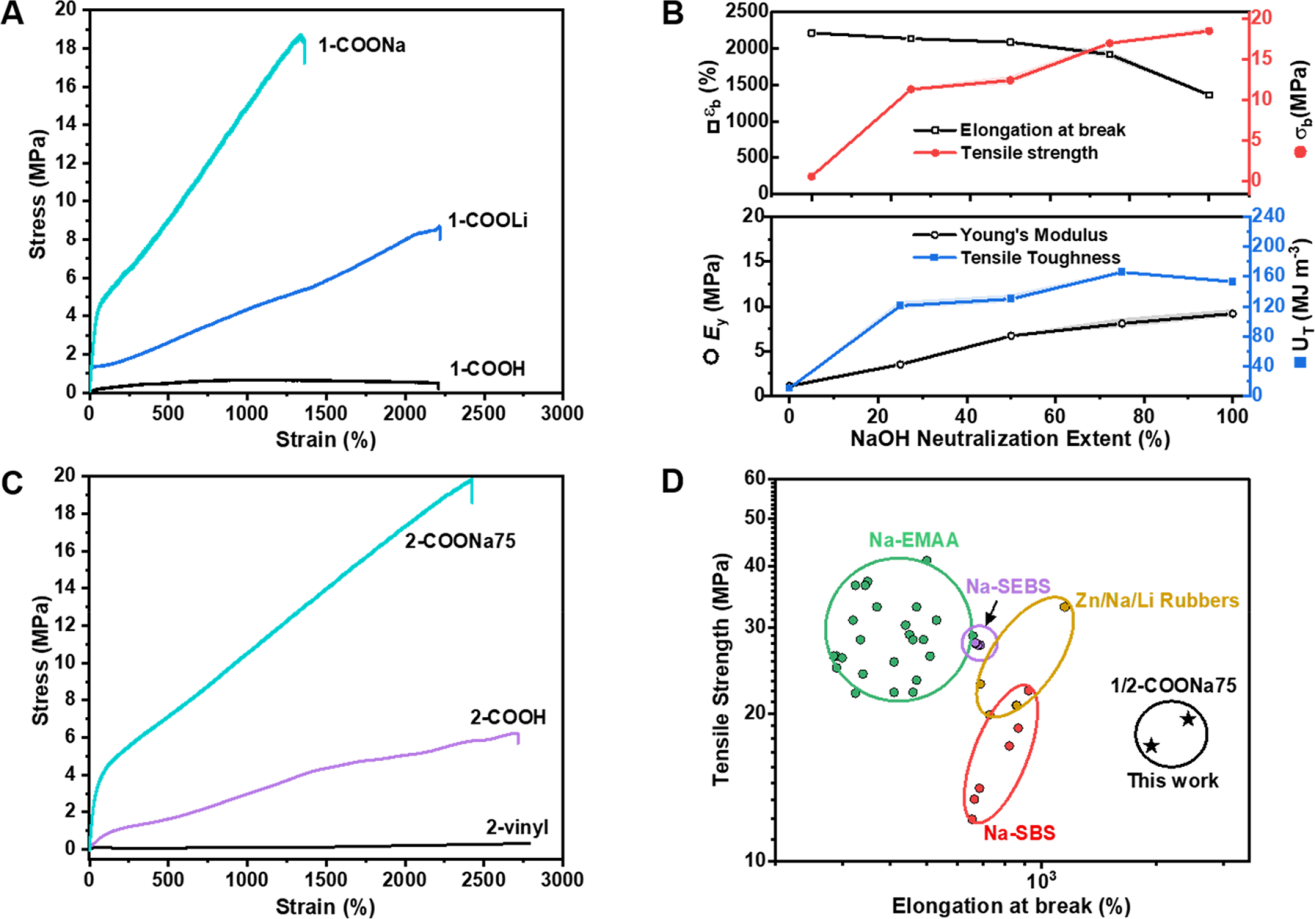
Tensile testing of polyester TPE ionomers. (A) Stress–strain curves (representative of three repeats) comparing **1**-COOH with 100% Na- or Li-neutralized derivatives. (B) Elongation at break (ε_b_), stress at break (σ_b_), Young’s modulus (*E*_y_), and tensile toughness (*U*_T_, calculated as the area under the stress–strain curve) as a function of the neutralization extent of **1**-COOH (0–100%). (C) Stress–strain curves for higher *M*_n_ triblock polyesters (**2**-vinyl = 100 kg mol^–1^; **1**-vinyl = 60 kg mol^–1^), comparing unmodified (**2**-vinyl), carboxylic acid-functionalized (**2**-COOH) and 75% Na-neutralized (**2**-COONa75). (D) Ashby plot (Table S5 for details): sodium ionomers of styrenic block copolymers (SBS, SEBS), random Na-/Zn-ethylene/methacrylic acid (EMAA) ionomers, and ionic rubbers.

Polymer films for mechanical testing were prepared using solution-casting methods and stored in a glovebox before testing (Figure S13 shows a sample’s mechanical performance after 1 month’s exposure to atmospheric moisture). One of the samples, polymer **1**-vinyl, that is, with *M*_n_ = 60 kg mol^–1^ and 21 wt % PE(v), failed to yield standalone films for mechanical testing. However, after installing the carboxylic acid functionalities, **1**-COOH formed transparent and colorless standalone films with the properties of a soft elastomer (Figure 3A and Table 1). The lithium sample, **1**-COOLi, shows a ∼16× increase in tensile strength while retaining comparable elongation at break to **1**-COOH. The sodium polymer, **1**-COONa, shows ∼35 × greater tensile strength and only a slight decrease in the elongation at break (1900%). Regardless of the functionality/ions present, all the polyesters remained transparent upon stretching (Figure S14). Testing the influence of partial neutralization showed that the tensile strength increased with the sodium content. It was found that 75% sodium carboxylates along the polymer backbone (**1**-COONa75) showed the optimum tensile toughness, as measured by the area under the stress–strain curves (Figure 3B and Figure S15). A high toughness >160 MJ m^–3^ was observed for this sample, and its stress at break reached ∼17 MPa (Table 1). The higher *M*_n_ triblock polymer, **2**-vinyl (100 kg mol^–1^) of comparable wt % PE(v), formed self-standing films. It also showed even better mechanical properties for **2**-COOH and **2**-COONa75 (Figure 3C). The sodium polyester, **2**-COONa75, showed the best properties, combining a high tensile strength, ∼ 20 MPa with an elongation at break >2400%, and a very high toughness, >260 MJ m^–3^. The improvement is attributed to ionic associations in the hard domain, limiting failure by chain pull-out and a lower molecular weight between entanglements, *M*_e_ in the PDL matrix (higher storage modulus plateau, vide infra).^53^

Common commercial TPEs are petroleum-based styrenic block copolymers (SIS, SEBS, and SBS) and make up the largest market share by volume. Literature reports of ionomeric styrenic block copolymers typically incorporate sodium sulfonate groups in the rigid polystyrene domains. Lead samples (**1/2**-COONa75) show properties that match or improve upon those of sodium sulfonated poly(styrene-*b*-butadiene-*b*-styrene) (Na-SBS) (Figure 3D and Table S5). For example, they show equivalent or higher tensile strengths (∼20 MPa) and greater extensibility (×2.6), leading to superior performance toughness (×3). One potential advantage of the polyester TPE ionomers besides end-of-life degradability (vide infra) is the significant control exerted during ionic group installation and the potential to install different groups using the thiol–ene reaction. **1/2**-COONa75 also shows a greater elongation at break (×6) than popular commercial ionomers based on random EMAA copolymers with Na- or Zn-carboxylate crosslinks, albeit with lower tensile strengths (<33 MPa). Other important TPE ionomers are based on rubbers such as Zn-carboxylate-crosslinked acrylonitrile–butadiene rubber, against which the new polymers show competitive performances (Figure 3D).

Interestingly, recent work incorporating Na-carboxylates in the soft polyisoprene matrix in SIS gave an impressive tensile strength (43 MPa) and elongation at break (2600%).^54^ This approach is a promising future direction as anhydride/epoxide ROCOP using functionalized commercial monomers should be able to readily yield soft polyesters incorporating ionic interactions.

So far, there are no other reports of all-polyester block polymer ionomers. To understand the impact of the current systems, the mechanical performance of **1/2**-COONa75 is compared against other block polyester-based TPEs, especially those where the hard domains are reinforced through stereocomplexation (Table 1). For example, Hillmyer and co-workers pioneered polyester elastomers featuring PDL midblocks and PLA/PLLA hard domains.^33,54^ Using the same soft-block polymer, **2**-COONa75 shows ∼3–4× greater toughness and achieves higher stress and elongation at break than the analogous materials featuring PLA or PLLA (A-blocks). The ionomer approach allows for straightforward tuning of the tensile strength from 1 to 20 MPa, without requiring new monomers or changes to polymer compositions/structures. The tensile stress of **2**-COONa75 approaches values reported for TPEs featuring polymenthide (PM, B-blocks, with stereocomplexed PLLA/PDLA, A-segments, Table 1)^38^ but falls short of those featuring poly(γ-methyl-ε caprolactone) (PγMCL) soft segments.^36^ This comparison highlights the significance of the soft-block polymer and its entanglement molecular weight in controlling TPE performances: PγMCL typically forms more entanglement soft segments than PDL. In the future, it should be possible to replace the PDL B-block in these new polyesters with alternative soft segments if higher TPE tensile strength were required. It is worth noting that compared to DL, these substituted lactone monomers are not yet commercially available. However, high-toughness TPEs (>445 MJ m^–3^) were recently reported with soft segments composed of random copolymers of commercial ε-caprolactone and δ-valerolactone (PCVL) with stereocomplexed PLA hard domains,^55^ although no indication of elastic recovery was given.

**Figure 4:**
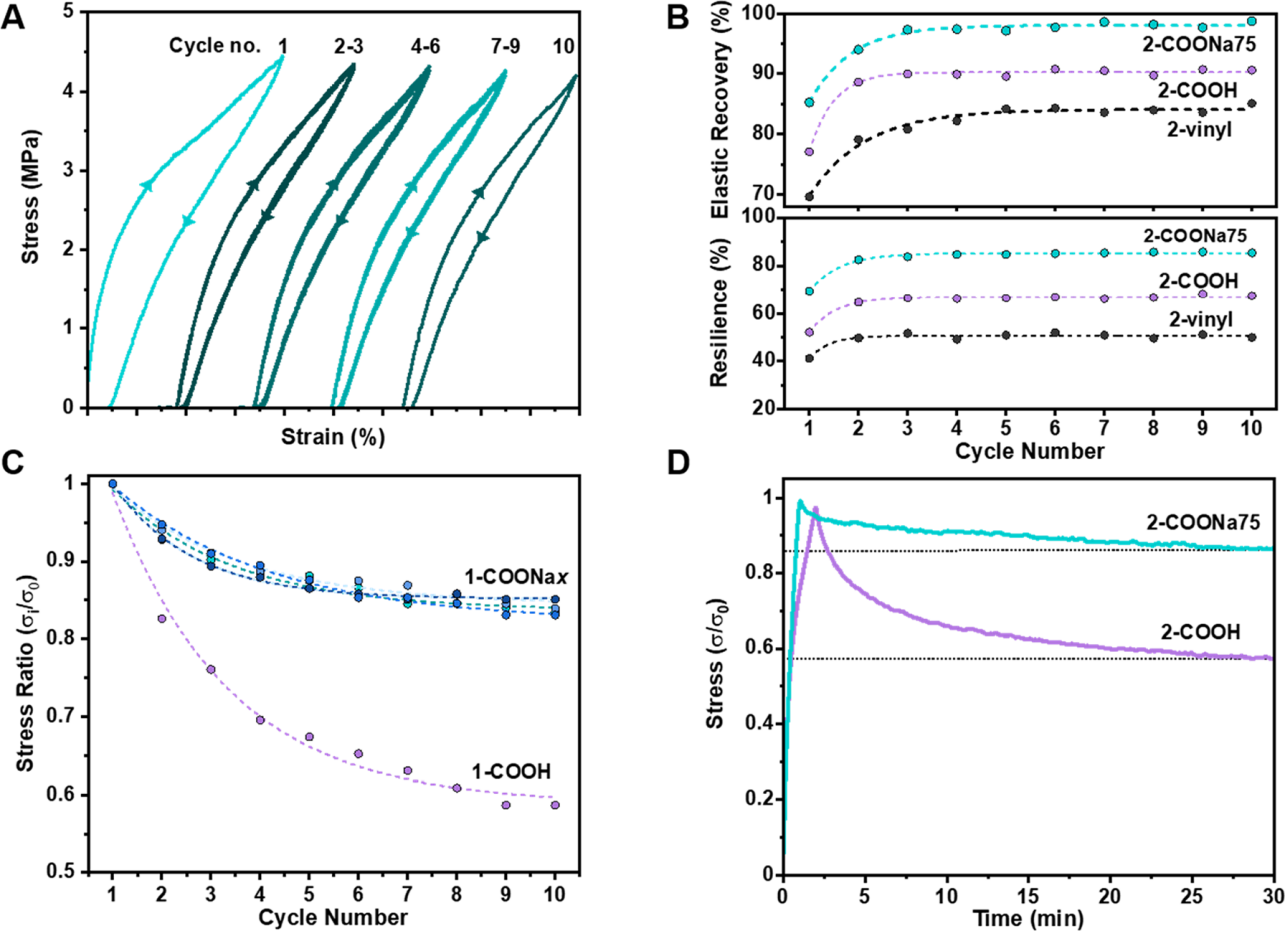
Elastic behavior. (A) Stretching–relaxing cycles of **2**-COONa75 to 200% strain. (B) Corresponding elastic recovery and resilience for each cycle. (C) Stress softening with cycle number; σ_0_ = maximum stress in the first cycle and σ_*i*_ = maximum stress in the subsequent cycle *i*. In **1**-COONa*x*, *x* = 25, 50, 75, and 100% neutralized. (D) Stress relaxation experiments at 200% applied strain; stress is normalized to the stress at time, *t* = 0 (σ_0_).

To better assess the elasticity of the ionomeric block-polyester TPEs, uniaxial cyclic tensile testing experiments were conducted. Dumbbell samples of **2**-COONa75 were stretched to 200% strain and then allowed to recover, a process repeated over 10 cycles (Figure 4A). The first cycle is different from the subsequent ones, a common phenomenon and one observed for all the block polyesters, attributed to the changes in the polymer microstructure under stress (Figure S16). Nonetheless, these changes are temporary since in all cases, the elastic recovery values [defined as (ε_max_ – ε_min_/ε_max_) × 100] are high at >80% (Figure S17). The highest elastic recovery values were observed for the sodium-, lithium-, or carboxylic acid-functionalized polymers. Indeed, the elastic recovery decreased in the order COOM (M = Li/Na) > COOH > vinyl (Figure 4B). These results can be rationalized by the soft PDL matrix being responsible for the extensibility and physical crosslinking from the hard domains, aiding recovery. Higher elastic recovery results from stronger phase separation in the COONa polymers, resulting in sharper interfaces (Figure 2D) and reinforcement of the hard chains either with hydrogen-bonding or stronger ionic interactions.^26,56,57^

The resilience or percentage of energy recovered during cyclic deformation was calculated as the ratio of the area under the unloading-to-loading curves. The TPE resilience increased with carboxylic acid functionalization and was even better with sodium neutralization (Figure 4B). For **2**-vinyl, the maximum stress, at 200% strain, decreased with each loading/unloading cycle such that after 7 cycles, the maximum stress plateaued at ∼60% of its original value. In contrast, **2**-COONa75 showed much less stress reduction and, after 7 cycles, retained 92% of its original value (Figure S18). In Figure 4C, the maximum stress, at 200% strain, is plotted for each cycle normalized to the original stress at cycle one (σ_i_/σ_0_) and gives a measure of stress softening. The sodium polymers, **1**-COONa*x*, where *x* = extent of ionization, show significantly less stress softening with the cycle number than pristine **1**-COOH. Interestingly, the effect appears to be independent of the extent of sodium neutralization.

As resilience and elastic recovery are important for TPE applications requiring load bearing and dynamic loading, time-dependent stress relaxation experiments were conducted. These experiments involved stretching dumbbell specimens of **2**-COOH and **2**-COONa75 to a constant strain of 200% and monitoring the reduction in stress with time (Figure 4D). At this strain, the stress was 1.1 and 3.0 MPa for **2**-COOH and **2**-COONa75, respectively. After 0.5 h, the stress had reduced to 50% for **2**-COOH, but it remained high at 85% for **2**-COONa75.

### Reprocessing and Degradation

**Figure 5:**
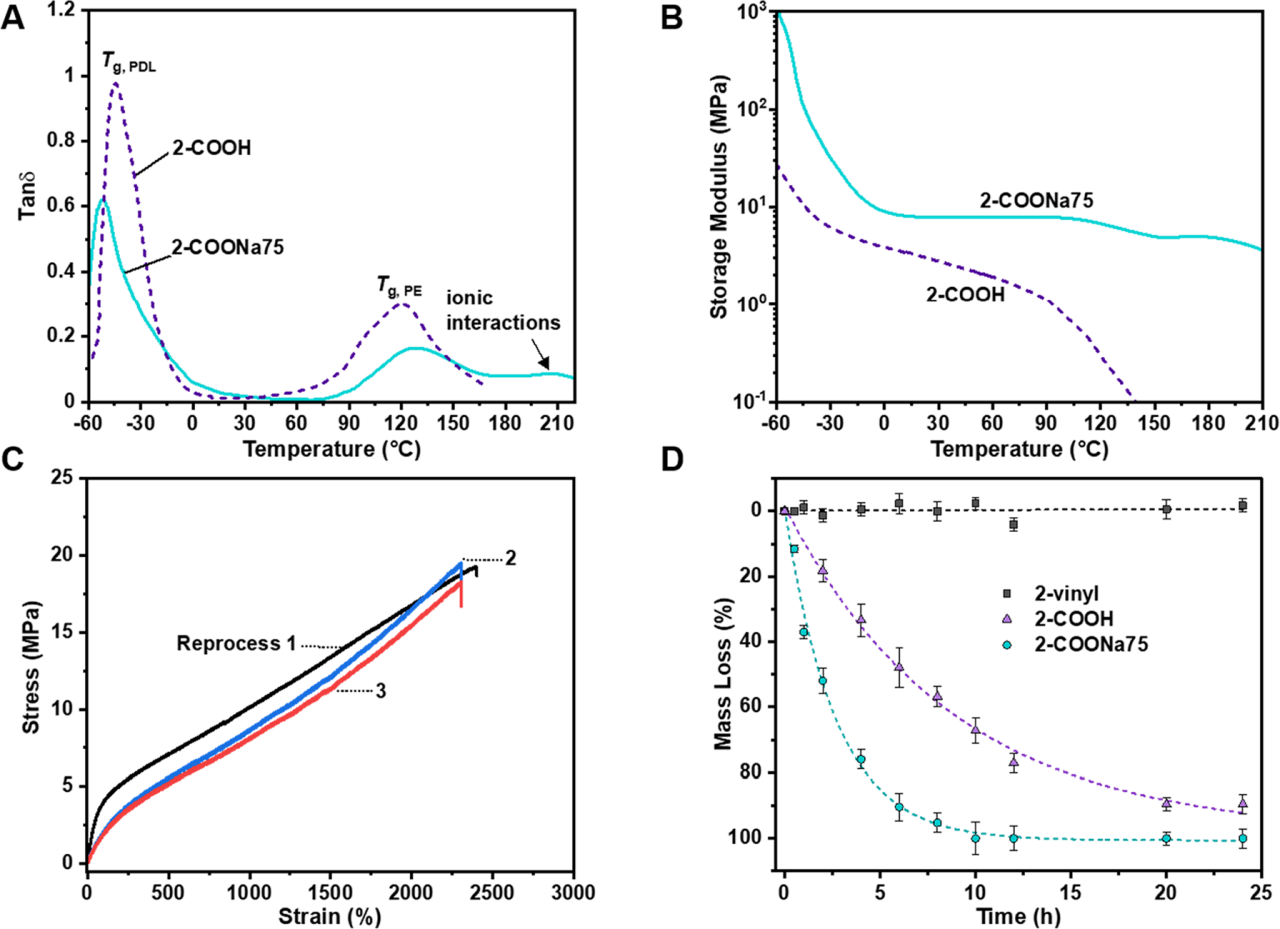
Thermal reprocessing of **2**-COONa75 and degradation studies. (A) Temperature dependence of tan δ (storage/loss modulus) for **2**-COOH and **2**-COONa75 measured by DMTA. (B) Temperature dependence of the storage modulus (MPa) for **2**-COOH and **2**-COONa75 measured by DMTA. (C) Stress–strain curves of **2**-COONa75 after repeated (×3) compression molding. (D) Degradation study in aqueous alkaline media (1 M NaOH, RT).

A critical future challenge for materials such as TPEs is to evaluate the potential for material reprocessing and thus recycling to equivalent products with retention of properties. These investigations are relevant in a circular economy for postconsumer waste and improved manufacturing efficiency by reprocessing polymer scraps and off-cuts generated during article fabrication. Many of the current commercial TPEs are surprisingly incompatible with such closed-loop recycling due to problems of either thermal instability or difficulties reproducing the original crystalline phase-separated structures.^58^ Natural rubber and other vulcanized thermosets cannot be recycled in this way. In these cases, down-cycling (*i.e.*, reuse in lower-value applications such as fillers in asphalt/composites) and combustion for heat recovery are currently the best options.^59^ Given its properties, the potential for thermal reprocessing **2**-COONa75 was investigated. First, its thermal processing window was assessed by dynamic mechanical thermal analysis (DMTA). The loss-to-storage modulus ratio (tanδ), over the temperature range from −60 to 220 °C, was compared for **2**-COONa75 and **2**-COOH (Figure 5A; see Figure S19 for storage and loss moduli).

Both data sets show a peak in tan δ at −52 °C, corresponding to the PDL glass transition, and subsequently, two higher-temperature transitions are observed for **2**-COONa75. The peak at 129 °C is consistent with the semiaromatic (PE) glass transition. The broad peak at ∼207 °C is attributed to transitions within the hard phase of sodium ion aggregates/clusters.^60−62^ It coincides with sufficient softening of the polymer to allow for reprocessing at temperatures around 200 °C. In contrast, **2**-COOH shows a much steeper drop in the elastic response (storage modulus) at the upper glass transition, and the sample yields at 160 °C. Consequently, as proof-of-concept recycling conditions, 2-COONa75 was reformulated at 200 °C and 20 MPa pressure. The tensile mechanical performance was determined after each reprocessing cycle. After the first and second cycles, the properties remained the same, within error, to the original sample (Figure 5B). After the third recycle, the polymer was slightly yellow, but its tensile strength remained almost the same (see Figure S20 for SEC after each thermal pressing step).

In future, these conditions will be fine-tuned to allow for increased (re)cycles. Another option will be to explore the polymers’ potential for chemical recycling. Given the solubility of the ionized polymers in polar media (THF/water mixtures), they should show faster water uptake and accelerated degradation.^29^ Thin films (200 μm thick) of **2**-vinyl, **2**-COOH, and **2**-COONa75 were cut into discs (16 mm diameter) and submerged into distilled water. After 1 h, mass increases due to the water uptakes of 11, 30, and 40%, respectively, were recorded (Figure S21). Degradation experiments under alkaline conditions (1 M NaOH, pH 14) were conducted by periodically removing the polymer discs and drying to constant mass (after substantial deterioration of the discs, solid particulates were isolated by centrifugation). Complete mass loss for **2**-COONa75 was observed after 10 h (Figure 5D). To differentiate this mass loss from the polymer dissolving in the aqueous media, SEC analysis of the residue isolated after removing the water revealed no detectable polymer. In contrast, the disc of **2**-vinyl remained completely intact after 24 h and no *M*_n_ loss was recorded by SEC. The rate of mass loss for **2**-COOH was notably slower than **2**-COONa75, and after 10 h, the residual polymer of **2**-COOH was still observable (through ∼1/2 *M*_n_). We previously demonstrated degradation under acidic conditions of triblock polyesters based on DL and PA/CHO to oligomers and small molecules.^41^ Here, ^1^H NMR spectroscopy (Figure S22) and LC-MS (Figure S23) of the water-solubilized degradation products for **2**-COONa75 suggested the presence of phthalic acid (*m*/*z* 165), the ring-opened diol of the functionalized epoxide (*m*/*z* 148), 2-hydroxydecanoic acid (*m*/*z* 187), and various smaller monomer repeat units (Table S6).

The toxicity of these degradation products should form the focus of future investigations. However, the methodologies and structures described in this proof-of-concept work are amenable to using other monomers, functional groups, and ions. In particular, the switchable catalysts function well with cyclic carbonates, thiiranes, carbon dioxide, or other heteroallenes, providing routes to other backbone polymer chemistries.^48^ Notably, there are opportunities to improve the biobased content and thus the potential for low-toxicity degradation products. For example, a current limitation of the work is the lack of a sustainable route to vCHO. Thus, future work might exploit the natural rigidity and alkene functionality afforded by terpene-derived anhydrides/epoxides such as limonene oxide (derived from citrus peel) in the hard domains.^63−65^ In contrast, DL is a biobased raw material (derived from castor oil), and routes to PA (currently petroleum-based) from corn stover have been reported.^66,67^ The metal-coordinating functional groups could be easily changed; for example, sulfonates, phosphates, or hydroxides are all compatible with the thiol–ene process.^46,68,69^ Changing the ions or metal salts affords access to different coordination chemistries and aggregates. Although in the context of both sustainability and cost considerations, the advantages to inexpensive, colorless, and low-toxicity sodium ions are hard to better.

## Conclusions

In conclusion, all-polyester ABA-type TPEs with controllable compositions and sodium/lithium ionization extents were prepared. The syntheses occurred in one pot using a single catalyst. The controlled ROP of ε-decalactone yielded hydroxyl telechelic PDL B-*block*, which initiated vinyl-cyclohexene oxide/PA ROCOP to install terminal alternating polyester A-blocks. The vinyl groups on the A-block polyesters were quantitatively transformed into carboxylic acid groups using a photoinitiated thiol–ene reaction. Subsequent neutralization of the acids with sodium or lithium hydroxide allowed tunable metalation to form lithium/sodium carboxylates. The synthetic methodology and polymer structures described here represent a straightforward means to “tune” the thermoplastic elastomeric properties over a wide range without requiring complex procedures, new monomers, or major polymer redesign. The ionized polymers significantly improved the tensile mechanical properties compared to either the unfunctionalized or carboxylic acid-substituted polyesters. For example, the stress at break increased 20–35-fold and toughness by 3–5×. The elongation at break and elastic recovery were highest (>90%) for the sodium polyesters, with high resilience during repeated deformation (85%). The lead sample, **2**-COONa75, showed properties closely aligned with commercial petrochemical TPEs and combined a high tensile strength ∼20 MPa, a high elasticity >2400%, and a very high toughness, >260 MJ m^–3^. All the polyesters, including ionized samples, can be thermally processed, and recycling experiments were conducted using **2**-COONa75 without the loss of properties. The formation of ionic aggregates is indicated in transitions observed by dynamic mechanical thermal analysis, and the influences of ions, functional groups, and ion separation will, in future, be systematically investigated. The methods are expected to be generalizable to many other monomer combinations, polymer structures, and ions. The work serves as the starting point for a new series of sustainable polymers and ionomers that span the property portfolio of the current elastomers.

## Supporting Information Available

Experimental and instrument details, polymer synthesis, characterization, and additional mechanical property measurements (PDF)

## References

[ref1] Zhu Y.; Romain C.; Williams C. K. Sustainable polymers from renewable resources. Nature 2016, 540, 354–362. 10.1038/nature21001. 27974763

[ref2] Coates G. W.; Getzler Y. D. Y. L. Chemical recycling to monomer for an ideal, circular polymer economy. Nat. Rev. Mater. 2020, 5, 501–516. 10.1038/s41578-020-0190-4.

[ref3] Schneiderman D. K.; Hillmyer M. A. 50th Anniversary Perspective: There Is a Great Future in Sustainable Polymers. Macromolecules 2017, 50, 3733–3749. 10.1021/acs.macromol.7b00293.

[ref4] Zhang X.; Fevre M.; Jones G. O.; Waymouth R. M. Catalysis as an Enabling Science for Sustainable Polymers. Chem. Rev. 2018, 118, 839–885. 10.1021/acs.chemrev.7b00329. 29048888

[ref5] Hong M.; Chen E. Y.-X. Chemically recyclable polymers: a circular economy approach to sustainability. Green Chem. 2017, 19, 3692–3706. 10.1039/c7gc01496a.

[ref6] Wang W.; Lu W.; Goodwin A.; Wang H.; Yin P.; Kang N.-G.; Hong K.; Mays J. W. Recent advances in thermoplastic elastomers from living polymerizations: Macromolecular architectures and supramolecular chemistry. Prog. Polym. Sci. 2019, 95, 1–31. 10.1016/j.progpolymsci.2019.04.002.

[ref7] Zanchin G.; Leone G. Polyolefin thermoplastic elastomers from polymerization catalysis: Advantages, pitfalls and future challenges. Prog. Polym. Sci. 2021, 113, 101342 10.1016/j.progpolymsci.2020.101342.

[ref8] Wang Z.; Yuan L.; Tang C. Sustainable Elastomers from Renewable Biomass. Acc. Chem. Res. 2017, 50, 1762–1773. 10.1021/acs.accounts.7b00209. 28636365

[ref9] Scheutz G. M.; Lessard J. J.; Sims M. B.; Sumerlin B. S. Adaptable Crosslinks in Polymeric Materials: Resolving the Intersection of Thermoplastics and Thermosets. J. Am. Chem. Soc. 2019, 141, 16181–16196. 10.1021/jacs.9b07922. 31525287

[ref10] Fortman D. J.; Brutman J. P.; De Hoe G. X.; Snyder R. L.; Dichtel W. R.; Hillmyer M. A. Approaches to Sustainable and Continually Recyclable Cross-Linked Polymers. ACS Sustainable Chem. Eng. 2018, 6, 11145–11159. 10.1021/acssuschemeng.8b02355.

[ref11] Ritchie R. O. The conflicts between strength and toughness. Nat. Mater. 2011, 10, 817–822. 10.1038/nmat3115. 22020005

[ref12] Yanagisawa Y.; Nan Y.; Okuro K.; Aida T. Mechanically robust, readily repairable polymers via tailored noncovalent cross-linking. Science 2018, 359, 72–76. 10.1126/science.aam7588. 29242235

[ref13] Winey K. I. Designing tougher elastomers with ionomers. Science 2017, 358, 449 10.1126/science.aap8114. 29074755

[ref14] Khare E.; Holten-Andersen N.; Buehler M. J. Transition-metal coordinate bonds for bioinspired macromolecules with tunable mechanical properties. Nat. Rev. Mater. 2021, 6, 421–436. 10.1038/s41578-020-00270-z.

[ref15] Wang W.; Zhang J.; Jiang F.; Wang X.; Wang Z. Reprocessable Supramolecular Thermoplastic BAB-Type Triblock Copolymer Elastomers with Enhanced Tensile Strength and Toughness via Metal–Ligand Coordination. ACS Appl. Polym. Mater. 2019, 1, 571–583. 10.1021/acsapm.8b00277.

[ref16] Li C.-H.; Wang C.; Keplinger C.; Zuo J.-L.; Jin L.; Sun Y.; Zheng P.; Cao Y.; Lissel F.; Linder C.; You X.-Z.; Bao Z. A highly stretchable autonomous self-healing elastomer. Nat. Chem. 2016, 8, 618–624. 10.1038/nchem.2492. 27219708

[ref17] Zheng N.; Xu Y.; Zhao Q.; Xie T. Dynamic Covalent Polymer Networks: A Molecular Platform for Designing Functions beyond Chemical Recycling and Self-Healing. Chem. Rev. 2021, 121, 1716–1745. 10.1021/acs.chemrev.0c00938. 33393759

[ref18] Chen Y.; Guan Z. Multivalent hydrogen bonding block copolymers self-assemble into strong and tough self-healing materials. Chem. Commun. 2014, 50, 10868–10870. 10.1039/c4cc03168g. 25090104

[ref19] Lai J.-C.; Li L.; Wang D.-P.; Zhang M.-H.; Mo S.-R.; Wang X.; Zeng K.-Y.; Li C.-H.; Jiang Q.; You X.-Z.; Zuo J.-L. A rigid and healable polymer cross-linked by weak but abundant Zn(II)-carboxylate interactions. Nat. Commun. 2018, 9, 2725 10.1038/s41467-018-05285-3. 30006515 PMC6045665

[ref20] Miwa Y.; Kurachi J.; Kohbara Y.; Kutsumizu S. Dynamic ionic crosslinks enable high strength and ultrastretchability in a single elastomer. Commun. Chem. 2018, 1, 5 10.1038/s42004-017-0004-9.

[ref21] Potaufeux J.-E.; Odent J.; Notta-Cuvier D.; Lauro F.; Raquez J.-M. A comprehensive review of the structures and properties of ionic polymeric materials. Polym. Chem. 2020, 11, 5914–5936. 10.1039/d0py00770f.

[ref22] SURLYN 8945 Ionomer Technical Data Sheet, https://www.dow.com (accessed 2022-02-25).

[ref23] Kajita T.; Tanaka H.; Noro A.; Matsushita Y.; Nozawa A.; Isobe K.; Oda R.; Hashimoto S. Extremely tough block polymer-based thermoplastic elastomers with strongly associated but dynamically responsive noncovalent cross-links. Polymer 2021, 217, 123419 10.1016/j.polymer.2021.123419.

[ref24] Enokida J. S.; Hu W.; Fang H.; Morgan B. F.; Beyer F. L.; Winter H. H.; Coughlin E. B. Modifying the Structure and Dynamics of Ionomers through Counterion Sterics. Macromolecules 2020, 53, 1767–1776. 10.1021/acs.macromol.9b02116.

[ref25] Aitken B. S.; Buitrago C. F.; Heffley J. D.; Lee M.; Gibson H. W.; Winey K. I.; Wagener K. B. Precision Ionomers: Synthesis and Thermal/Mechanical Characterization. Macromolecules 2012, 45, 681–687. 10.1021/ma202304s.

[ref26] Kawana S.; Nakagawa S.; Nakai S.; Sakamoto M.; Ishii Y.; Yoshie N. Interphase synergistic effects of dynamic bonds in multiphase thermoplastic elastomers. J. Mater. Chem. A 2019, 7, 21195–21206. 10.1039/c9ta07522d.

[ref27] Perry S. L.; Sing C. E. 100th Anniversary of Macromolecular Science Viewpoint: Opportunities in the Physics of Sequence-Defined Polymers. ACS Macro Lett. 2020, 9, 216–225. 10.1021/acsmacrolett.0c00002. 35638672

[ref28] Hayashi M.; Obara H.; Miwa Y. Design and basic properties of polyester vitrimers combined with an ionomer concept. Mol. Syst. Des. Eng. 2021, 6, 234–241. 10.1039/d1me00002k.

[ref29] Han S.-I.; Yoo Y.; Kim D. K.; Im S. S. Biodegradable Aliphatic Polyester Ionomers. Macromol. Biosci. 2004, 4, 199–207. 10.1002/mabi.200300095. 15468209

[ref30] Johnston P.; Adhikari R. Synthesis, properties and applications of degradable ionomers. Eur. Polym. J. 2017, 95, 138–160. 10.1016/j.eurpolymj.2017.08.009.

[ref31] Wanamaker C. L.; O’Leary L. E.; Lynd N. A.; Hillmyer M. A.; Tolman W. B. Renewable-Resource Thermoplastic Elastomers Based on Polylactide and Polymenthide. Biomacromolecules 2007, 8, 3634–3640. 10.1021/bm700699g. 17960909

[ref32] Hillmyer M. A.; Tolman W. B. Aliphatic Polyester Block Polymers: Renewable, Degradable, and Sustainable. Acc. Chem. Res. 2014, 47, 2390–2396. 10.1021/ar500121d. 24852135

[ref33] Martello M. T.; Schneiderman D. K.; Hillmyer M. A. Synthesis and Melt Processing of Sustainable Poly(ε-decalactone)-block-Poly(lactide) Multiblock Thermoplastic Elastomers. ACS Sustainable Chem. Eng. 2014, 2, 2519–2526. 10.1021/sc500412a.

[ref34] Martello M. T.; Hillmyer M. A. Polylactide–Poly(6-methyl-ε-caprolactone)–Polylactide Thermoplastic Elastomers. Macromolecules 2011, 44, 8537–8545. 10.1021/ma201063t.

[ref35] Schneiderman D. K.; Hill E. M.; Martello M. T.; Hillmyer M. A. Poly(lactide)-block-poly(ε-caprolactone-co-ε-decalactone)-block-poly(lactide) copolymer elastomers. Polym. Chem. 2015, 6, 3641–3651. 10.1039/c5py00202h.

[ref36] Watts A.; Kurokawa N.; Hillmyer M. A. Strong, Resilient, and Sustainable Aliphatic Polyester Thermoplastic Elastomers. Biomacromolecules 2017, 18, 1845–1854. 10.1021/acs.biomac.7b00283. 28467049

[ref37] Batiste D. C.; Meyersohn M. S.; Watts A.; Hillmyer M. A. Efficient Polymerization of Methyl-ε-Caprolactone Mixtures To Access Sustainable Aliphatic Polyesters. Macromolecules 2020, 53, 1795–1808. 10.1021/acs.macromol.0c00050.

[ref38] Wanamaker C. L.; Bluemle M. J.; Pitet L. M.; O’Leary L. E.; Tolman W. B.; Hillmyer M. A. Consequences of Polylactide Stereochemistry on the Properties of Polylactide-Polymenthide-Polylactide Thermoplastic Elastomers. Biomacromolecules 2009, 10, 2904–2911. 10.1021/bm900721p. 19775147

[ref39] Worch J. C.; Prydderch H.; Jimaja S.; Bexis P.; Becker M. L.; Dove A. P. Stereochemical enhancement of polymer properties. Nat. Rev. Chem. 2019, 3, 514–535. 10.1038/s41570-019-0117-z.

[ref40] Schneiderman D. K.; Hillmyer M. A. Aliphatic Polyester Block Polymer Design. Macromolecules 2016, 49, 2419–2428. 10.1021/acs.macromol.6b00211.

[ref41] Zhu Y.; Radlauer M. R.; Schneiderman D. K.; Shaffer M. S. P.; Hillmyer M. A.; Williams C. K. Multiblock Polyesters Demonstrating High Elasticity and Shape Memory Effects. Macromolecules 2018, 51, 2466–2475. 10.1021/acs.macromol.7b02690.

[ref42] Gregory G. L.; Sulley G. S.; Carrodeguas L. P.; Chen T. T. D.; Santmarti A.; Terrill N. J.; Lee K.-Y.; Williams C. K. Triblock polyester thermoplastic elastomers with semi-aromatic polymer end blocks by ring-opening copolymerization. Chem. Sci. 2020, 11, 6567–6581. 10.1039/d0sc00463d. 34094122 PMC8159401

[ref43] Yi N.; Chen T. T. D.; Unruangsri J.; Zhu Y.; Williams C. K. Orthogonal functionalization of alternating polyesters: selective patterning of (AB)n sequences. Chem. Sci. 2019, 10, 9974–9980. 10.1039/c9sc03756j. 32015813 PMC6968736

[ref44] Sanford M. J.; Van Zee N. J.; Coates G. W. Reversible-deactivation anionic alternating ring-opening copolymerization of epoxides and cyclic anhydrides: access to orthogonally functionalizable multiblock aliphatic polyesters. Chem. Sci. 2018, 9, 134–142. 10.1039/c7sc03643d. 29629081 PMC5868299

[ref45] Wang Y.; Fan J.; Darensbourg D. J. Construction of Versatile and Functional Nanostructures Derived from CO2-based Polycarbonates. Angew. Chem., Int. Ed. 2015, 54, 10206–10210. 10.1002/anie.201505076. 26177634

[ref46] Darensbourg D. J.; Tsai F.-T. Postpolymerization Functionalization of Copolymers Produced from Carbon Dioxide and 2-Vinyloxirane: Amphiphilic/Water-Soluble CO2-Based Polycarbonates. Macromolecules 2014, 47, 3806–3813. 10.1021/ma500834r.

[ref47] Liang X.; Tan F.; Zhu Y. Recent Developments in Ring-Opening Copolymerization of Epoxides With CO2 and Cyclic Anhydrides for Biomedical Applications. Front. Chem. 2021, 9, 647245 10.3389/fchem.2021.647245. 33959588 PMC8093832

[ref48] Deacy A. C.; Gregory G. L.; Sulley G. S.; Chen T. T. D.; Williams C. K. Sequence Control from Mixtures: Switchable Polymerization Catalysis and Future Materials Applications. J. Am. Chem. Soc. 2021, 143, 10021–10040. 10.1021/jacs.1c03250. 34190553 PMC8297863

[ref49] Romain C.; Zhu Y.; Dingwall P.; Paul S.; Rzepa H. S.; Buchard A.; Williams C. K. Chemoselective Polymerizations from Mixtures of Epoxide, Lactone, Anhydride, and Carbon Dioxide. J. Am. Chem. Soc. 2016, 138, 4120–4131. 10.1021/jacs.5b13070. 27003333

[ref50] Sulley G. S.; Gregory G. L.; Chen T. T. D.; Peña Carrodeguas L.; Trott G.; Santmarti A.; Lee K.-Y.; Terrill N. J.; Williams C. K. Switchable Catalysis Improves the Properties of CO2-Derived Polymers: Poly(cyclohexene carbonate-b-ε-decalactone-b-cyclohexene carbonate) Adhesives, Elastomers, and Toughened Plastics. J. Am. Chem. Soc. 2020, 142, 4367–4378. 10.1021/jacs.9b13106. 32078313 PMC7146851

[ref51] Olsén P.; Borke T.; Odelius K.; Albertsson A. C. ε-Decalactone: a thermoresilient and toughening comonomer to poly(L-lactide). Biomacromolecules 2013, 14, 2883 10.1021/bm400733e. 23815125

[ref52] Navratil M.; Eisenberg A. Ion Clustering and Viscoelastic Relaxation in Styrene-Based Ionomers. III. Effect of Counterions, Carboxylic Groups, and Plasticizers. Macromolecules 1974, 7, 84–89. 10.1021/ma60037a017.

[ref53] Tong J.-D.; Jerôme R. Dependence of the Ultimate Tensile Strength of Thermoplastic Elastomers of the Triblock Type on the Molecular Weight between Chain Entanglements of the Central Block. Macromolecules 2000, 33, 1479–1481. 10.1021/ma990404f.

[ref54] Lee S.; Lee K.; Kim Y.-W.; Shin J. Preparation and Characterization of a Renewable Pressure-Sensitive Adhesive System Derived from ε-Decalactone, l-Lactide, Epoxidized Soybean Oil, and Rosin Ester. ACS Sustainable Chem. Eng. 2015, 3, 2309–2320. 10.1021/acssuschemeng.5b00580.

[ref55] Zhao W.; Li C.; Yang X.; He J.; Pang X.; Zhang Y.; Men Y.; Chen X. One-Pot Synthesis of Supertough, Sustainable Polyester Thermoplastic Elastomers Using Block-Like, Gradient Copolymer as Soft Midblock. CCS Chem. 2021, 4, 1522–1531. 10.31635/ccschem.021.202100897.

[ref56] Sheiko S. S.; Dobrynin A. V. Architectural Code for Rubber Elasticity: From Supersoft to Superfirm Materials. Macromolecules 2019, 52, 7531–7546. 10.1021/acs.macromol.9b01127.

[ref57] Cordier P.; Tournilhac F.; Soulié-Ziakovic C.; Leibler L. Self-healing and thermoreversible rubber from supramolecular assembly. Nature 2008, 451, 977–980. 10.1038/nature06669. 18288191

[ref58] Drobny J. G. 16-Recycling of Thermoplastic Elastomers. In Handbook of Thermoplastic Elastomers, Drobny J. G., Ed.; William Andrew Publishing: Norwich, NY, 2007; pp 317–318.

[ref59] Sienkiewicz M.; Kucinska-Lipka J.; Janik H.; Balas A. Progress in used tyres management in the European Union: A review. Waste Manag. 2012, 32, 1742–1751. 10.1016/j.wasman.2012.05.010. 22687707

[ref60] Eisenberg A.; Hird B.; Moore R. B. A new multiplet-cluster model for the morphology of random ionomers. Macromolecules 1990, 23, 4098–4107. 10.1021/ma00220a012.

[ref61] Basu D.; Das A.; Stöckelhuber K. W.; Jehnichen D.; Formanek P.; Sarlin E.; Vuorinen J.; Heinrich G. Evidence for an in Situ Developed Polymer Phase in Ionic Elastomers. Macromolecules 2014, 47, 3436–3450. 10.1021/ma500240v.

[ref62] Salaeh S.; Das A.; Wießner S. Design and fabrication of thermoplastic elastomer with ionic network: A strategy for good performance and shape memory capability. Polymer 2021, 223, 123699 10.1016/j.polymer.2021.123699.

[ref63] Della Monica F.; Kleij A. W. From terpenes to sustainable and functional polymers. Polym. Chem. 2020, 11, 5109–5127. 10.1039/d0py00817f.

[ref64] Carrodeguas L. P.; Chen T. T. D.; Gregory G. L.; Sulley G. S.; Williams C. K. High elasticity, chemically recyclable, thermoplastics from bio-based monomers: carbon dioxide, limonene oxide and ε-decalactone. Green Chem. 2020, 22, 8298–8307. 10.1039/d0gc02295k.

[ref65] Chen T. T. D.; Carrodeguas L. P.; Sulley G. S.; Gregory G. L.; Williams C. K. Bio-based and Degradable Block Polyester Pressure-Sensitive Adhesives. Angew. Chem., Int. Ed. 2020, 59, 23450–23455. 10.1002/anie.202006807. PMC7756385 32886833

[ref66] Thiyagarajan S.; Genuino H. C.; Śliwa M.; van der Waal J. C.; de Jong E.; van Haveren J.; Weckhuysen B. M.; Bruijnincx P. C. A.; van Es D. S. Substituted Phthalic Anhydrides from Biobased Furanics: A New Approach to Renewable Aromatics. ChemSusChem 2015, 8, 3052–3056. 10.1002/cssc.201500511. 26235971

[ref67] Giarola S.; Romain C.; Williams C. K.; Hallett J. P.; Shah N. Techno-economic assessment of the production of phthalic anhydride from corn stover. Chem. Eng. Res. Des. 2016, 107, 181–194. 10.1016/j.cherd.2015.10.034.

[ref68] Zhang Y.-Y.; Wu G.-P.; Darensbourg D. J. CO2-Based Block Copolymers: Present and Future Designs. Trends Chem. 2020, 2, 750–763. 10.1016/j.trechm.2020.05.002.

[ref69] Lowe A. B. Thiol-ene “click” reactions and recent applications in polymer and materials synthesis. Polym. Chem. 2010, 1, 17–36. 10.1039/b9py00216b.

