# Supplementary material for: Exploiting Sodium Coordination in Alternating Monomer Sequences to Toughen Degradable Block Polyester Thermoplastic Elastomers
Source: Macromolecules. 2022 Mar 3;55(6):2290–9. doi: 10.1021/acs.macromol.2c00068 (PMC9084597; doi:10.1021/acs.macromol.2c00068)
Supplement: Supplementary file 1 — ma2c00068_si_001.pdf [file ma2c00068_si_001.pdf]

Supplementary Information for:

Exploiting Sodium Coordination in Alternating Monomer Sequences to Toughen Degradable Block Polyester Thermoplastic Elastomers

Georgina L. Gregory,* Charlotte K. Williams*

Table of Contents

Methods 2

Experimental Details 3

Scheme 1. ROCOP mechanism and heterodinuclear catalyst used in this work. 4

Figure S1. Polymer End-group Analysis.. 5

Figure S2. DOSY NMR spectrum ($CDCl_3$). 5

Figure S3. Size Exclusion Chromatography (SEC) Traces. 6

Figure S4. ^{1}H NMR spectrum ($CDCl_3$) of 1-vinyl. 6

Table S1. Polymer synthesis conditions. 7

Figure S5. ^{1}H NMR Spectra ($CDCl_3$) 7

Figure S6. $^{13}C\{^1H\}$ NMR Spectra ($CDCl_3$) of 1-vinyl and 1-COOH. 8

Figure S7. SEC Traces after modification with 3-mercaptopropionic acid (MPA) 8

Figure S8. ATR-FTIR Spectra (thin-film) of 1-COONa*x* 9

Figure S9. ^{1}H, ^{7}Li and ^{23}Na NMR Spectra (THF/D_2O) of 1-COOLi and 1-COONa. 9

Table S2. ICP Analysis of polymer samples. 9

Figure S10. SEC Trace of 1-COONa. 10

Figure S11. DSC traces of polymer films. 10

Figure S12. TGA analyses of polymers. 10

Table S3. Characterization of block copolyester phase morphology by SAXS 11

Figure S13. Influence of moisture content on mechanical properties. 11

Figure S14. Photo showing the transparency of block polyester TPE ionomers. 11

Figure S15. Stress-Strain curves showing repeat measurements. 12

Figure S16. Loading-unloading curves for polymers repeatably stretched to 200% strain.. 13

Figure S17. Elastic recovery. 14

Figure S18. Stress Softening Behaviour during Cyclic Tensile Testing. 14

Figure S19. Temperature Dependence of Storage (*E*') and Loss (*E*'') Moduli. 15

Table S4. Summary of Tensile Mechanical and Thermal data. 15

Figure S20. Thermal reprocessing. 15

Table S5: Examples of Ionic TPEs. 16

Figure S21. Water Uptake Experiments. 16

Figure S22. Degradation Products of 2-COONa75 in alkaline media. 17

Figure S23. LC-Mass spectrometry of degradation products from 2-COONa75.. 18

Table S6: Proposed Degradation Products of 2-COONa75 in alkaline media. 18

References 19

Materials

ε-Decalactone (DL) (Sigma-Aldrich, 98% purity) was dried over CaH_2 and fractionally distilled before being degassed by bubbling N_2 and stored in a glovebox. 4-Vinyl cyclohexene oxide (vCHO) (Acros) was dried over CaH_2 and purified by fractional distillation. 1,4-Benzenedimethanol, 1,4-BDM, (Alfa Aesar) was recrystallized (three times) from dry toluene (purified by SPS and stored over 4 Å molecular sieves) (~2.5 g in 100 mL hot toluene). Phthalic anhydride (PA) (Sigma Aldrich, anhydrous, 98% purity) was stirred in dry toluene (purified by SPS) for 12 h. The PA dissolves in the toluene solvent, and any insoluble phthalic acid impurity is removed by cannula filtration and removal of the toluene *in vacuo*. The resulting white powder was recrystallized from anhydrous chloroform (Sigma-Aldrich, amylene stabilizers) before being sublimed under vacuum at 80 °C and stored in a glovebox. Toluene was obtained from an SPS (< 4 ppm H_2O) and stored over 4 Å molecular sieves for at least 24 h before use. The catalyst, $[LZnMg(C_6F_5)_2]$, was prepared according to the literature procedure.[1]

Methods

NMR Spectroscopy: ^1H, ^7Li, ^{23}Na, ^{31}P and $^{13}C\{^1H\}$ NMR spectra were recorded on a Bruker Avance III HD 400 MHz spectrometer. DOSY spectra were recorded on Bruker Avance III HD 500 MHz spectrometer.

Size Exclusion Chromatography (SEC): Polymers (2-10 mg), dissolved in 1 mL HPLC grade THF, were syringe filtered through 2 µm filters before being injected into a Shimadzu LC-20AD GPC instrument, with two PSS SDV 5 µm linear M columns heated to 30 °C. HPLC grade THF was used as the eluent at a flow rate of 1.0 mL min^{-1}. RI and UV detectors were calibrated using narrow molecular weight polystyrene standards. Shimadzu GPC post-run program was used to analyze the data.

Differential Scanning Calorimetry (DSC): These were recorded for purified polymer samples as powders or films on a Mettler Toledo DSC3 Star calorimeter under a nitrogen flow (80 mL min^{-1}). Samples were first heated to 200 °C and held at that temperature for 5 minutes to remove any thermal history, before heating and cooling from -80 to 200 °C at a rate of 10 °C min^{-1}. Glass transition temperatures (T_g) were determined from the midpoint of the transition in the third heating curve.

Thermogravimetric Analysis (TGA): These were measured on a Mettler-Toledo Ltd TGA 1 system. Powder polymer samples were heated from 30 to 500 °C, at a rate of 10 °C min^{-1}, under a nitrogen flow (100 mL min^{-1}).

Tensile Testing: Dumbbell specimens were cut according to ISO standard 527-2, specimen type 5B with a Zwick ZCP020 cutting press (length= 35 mm, gauge length = 10 mm, width = 2 mm). Monotonic uniaxial extension experiments were carried out on a Shimadzu EZ-LZ Universal testing instrument at an extension rate of 10 mm min^{-1}. An external camera was used to calculate the Young's Modulus, E_y within the 0.025-0.25% strain region. 10 Specimens were tested for each material. Cyclic tensile tests were conducted to 200% strain at a rate of 10 mm min^{-1}. 10 Cycles were measured for each specimen, three specimens for each sample.

Thermal Press: Polymer films were pressed or reprocessed using the Carver mini CH CE Press (5420CE.4010C00) with heated plates and a hydraulic press. Powder samples were heated to 200 °C, before being pressed at 3000 psi for 15 minutes. Samples were then immediately removed and allowed to cool to room temperature.

ATR-FTIR Spectroscopy: FTIR spectra were recorded of polymer thin films on a Bruker Tensor 27 spectrophotometer, using 32 scans from 4000 to 600 cm^{-1}, at a resolution of 4 cm^{-1}.

Dynamic Mechanical Thermal Analysis (DMTA): These were recorded of polymer films on a TA instruments RSA-G2 Solids Analyser in tension mode. A pre-load force of 0.05 N was

applied to the rectangular specimens. The samples were strained to 0.1% at a frequency of 1 Hz and heated between -80 and 220 °C, at a rate of 3 °C min^{-1}.

Small Angle X-ray Scattering (SAXS). Polymer films were prepared by solvent casting from THF (vinyl- and COOH- substituted) or THF/water (ionomers) and drying at room temperature followed by in a vacuum oven at 80 °C for at least 72 h or until no residual solvent was observed by NMR or TGA analysis. This was analogous to the preparation of films for mechanical testing. Films were then submitted to Harwell Diamond Light Source in a solid sample grid for SAXS analysis (DL-SAXS, P38 instrument). Scans (3 × 5 min) were conducted at camera lengths of 4.5 and 1 m, beam energy = 9.2 keV (using the Ga MetalJet). SAXS curves reported are an average of the 3 scans measured from data collected at 1 m. Samples were not annealed prior to testing to reflect experimental conditions of tensile testing.[2,3]

Experimental Details

General Polymerization Procedure: In a glovebox, ε-DL (7.2 mL, 41 mmol, 2000 equiv.) was added to a Schlenk flask charged with a stirrer bar. 1,4-BDM (11 mg, 0.082 mmol, 4 equiv.) followed by toluene (17 mL), vCHO (3.1 mL, 25 mmol, 1200 equiv.) and then $[LZn_2Mg_2(C_6F_5)_2]$ (20 mg, 0.0205 mmol, 1 equiv.) were then added. The Schlenk tube was sealed and transferred to an oil bath, preheated to 80 °C. After 1 hour, the viscous solution was quenched by cooling in an ice-water bath. Once the solution was cool, an aliquot was taken under a nitrogen atmosphere for NMR analysis to quantify the ε-DL conversion to PDL. PA (2.4 g, 16.4 mmol, 400 equiv.) was then added to the solution in a glovebox. The reaction mixture was returned to the hot oil bath (100 °C), and after 36 h, the PA conversion was determined by NMR analysis of an aliquot. The crude reaction mixture was purified by precipitation into methanol (~500 mL). The white polymer was separated and dried under vacuum to remove all solvents (8.2 g).

General Polymer Post-functionalization Procedure: Polymer (1 g) was dissolved in degassed THF (10 mL) in an air-tight vial, charged with a magnetic stirrer bar. To the solution was added, 3-mercaptopropionic acid (128 µL, 1.5 mmol, 2 equiv.) followed by DMPA (38 mg, 0.15 mmol, 0.2 equiv.). The solution was then stirred, under UV light for 0.5 h, before being poured into a large excess of methanol. The polymer was isolated as a white substance (0.94 g, 84%).

Polymer Neutralization Procedure: To a rapidly stirring solution of polymer (0.2 g) dissolved in THF (2 mL) was added NaOH or LiOH as an aqueous stock solution. The reaction was stirred for 10 minutes, and the pH was monitored. Excess NaOH or LiOH was removed by dialysis against water.

Polymer End-Group Test: Following a literature procedure,[4] to polymer (40 mg) dissolved in $CDCl_3$ (0.4 mL) was added 40 µL of solution containing $Cr(acac)_3$ (5.5 mg) and internal standard, bisphenol A (400 mg) in pyridine (10 mL) followed by 40 µL of 2-chloro-4,4,5,5-tetramethyl dioxaphospholane. The $^{31}P\{^1H\}$ NMR spectrum was obtained, and comparisons were drawn between the resonances of signals for the constituent polymers.

Degradation Experiments: Polymer films prepared by solvent casting (thickness ~ 200 µm) were cut into discs (diameter = 16 mm). The discs were then submerged in distilled water. Samples were removed periodically, patted dry, and the mass increase recorded. No degradation of the samples was observed over 1 week in distilled water. Degradation experiments were subsequently conducted on discs submerged in aqueous alkaline solution (1 M NaOH, pH 14). Samples were removed periodically, dried to constant mass and the mass recorded as a percentage of the original dry mass (mass loss). Samples were also taken of the discs for SEC analysis. For **2**-COONa75, the solid was isolated by centrifugation after

sample discs disintegrated into particulates. After complete mass loss, the aqueous solution was removed in vacuo, and the resulting residue was analyzed by ^{1}H NMR, SEC, and mass spectrometry.

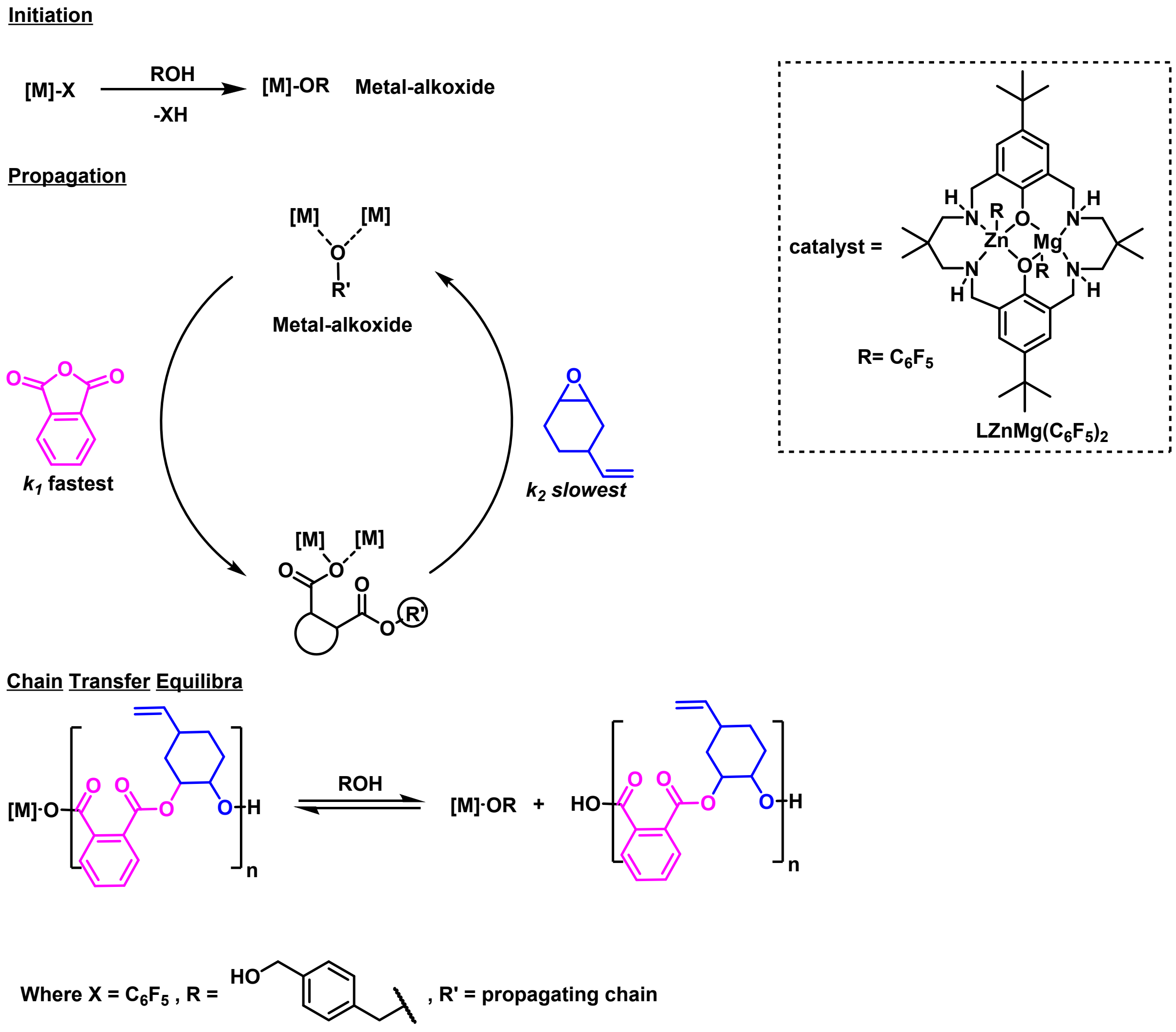

Scheme 1. ROCOP mechanism and heterodinuclear catalyst used in this work. As reported previously with this catalyst for ROCOP, no ether linkages from ROP of the epoxide are observed. The polymerization is highly selective for alternating PA/vCHO ROCOP.

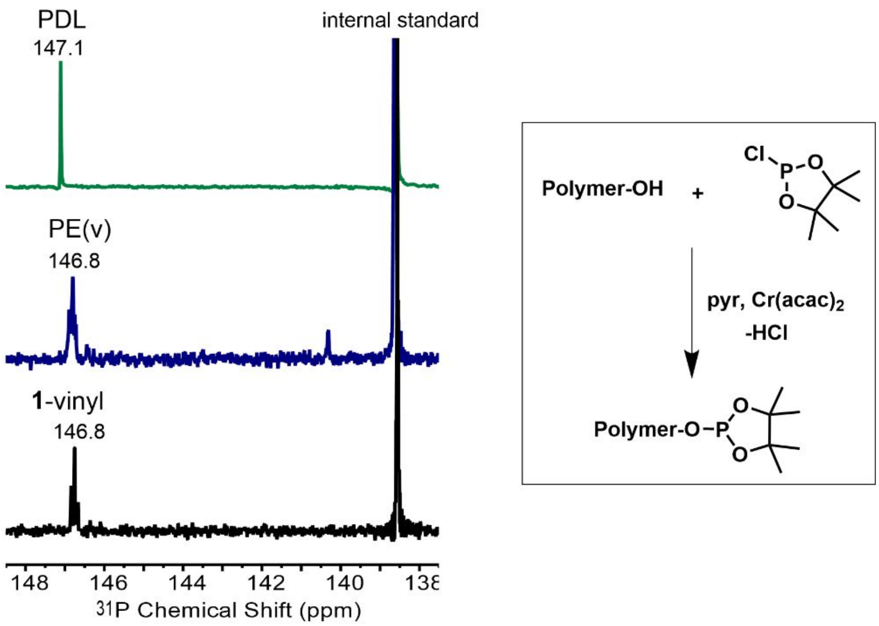

Figure S1. Polymer End-group Analysis. $^{31}P\{^{1}H\}$ NMR spectra ($CDCl_3$) after reaction of polymer hydroxyl end groups with 2-chloro-4,4,5,5-tetramethyldioxaphospholane (see experimental details above) showing PE(v) (146.7-146.8 ppm), PDL (147.1 ppm) and **1**-vinyl (146.7-146.8 ppm). The reaction with bisphenol A (BPA) is used as an internal standard at 138.6 ppm. The PE(v) shows several different resonances due to different head-tail configurations due to the lack of regioselectivity and stereoselectivity in the polymerization.

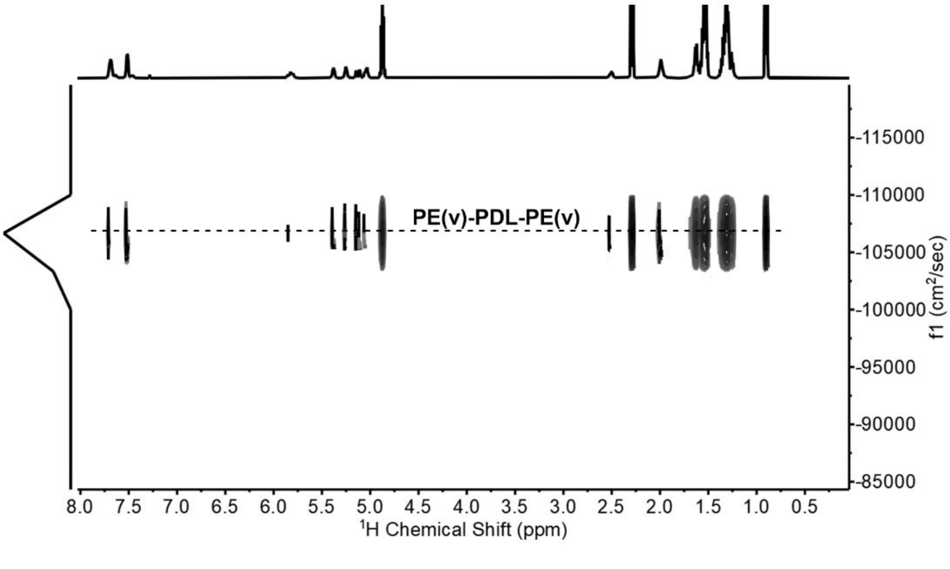

Figure S2. DOSY NMR spectrum ($CDCl_3$). A single diffusion coefficient is observed for the triblock polymer PE(v)-PDL-PE(v) (**1**-vinyl), supporting the connection of the blocks.

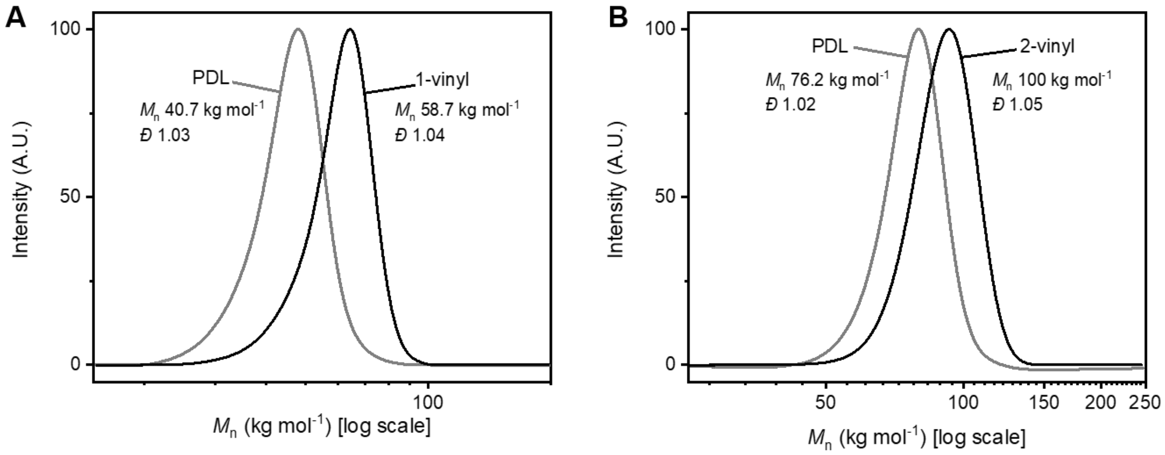

Figure S3. Size Exclusion Chromatography (SEC) Traces. A: Purified triblock polymer, **1**-vinyl and the PDL midblock measured from an aliquot of the reaction mixture taken before adding PA. B: PDL midblock and purified triblock polymer, **2**-vinyl. All traces were measured in THF eluent, with an RI detector and molecular weight values are versus narrow polystyrene standards.

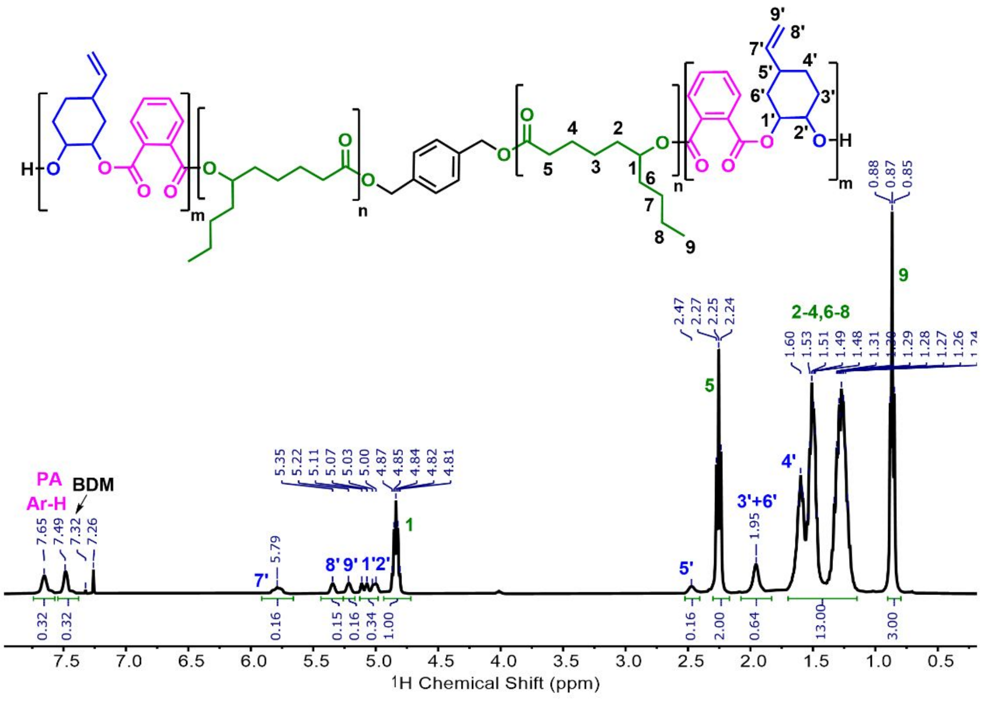

Figure S4. ^{1}H NMR spectrum ($CDCl_3$) of 1-vinyl. The wt% of PE(v) was determined by relative integration of the PDL signal at 4.85 ppm and PE(v) at 5.79 ppm.

Table S1. Polymer synthesis conditions.[a]

Sample	DL Conv. (%)[b]	PA Conv. (%)[c]	mol% MPA	$M_{n,theo}$ (kg mol^{-1})[d]	$M_{n,SEC}$ (kg mol^{-1}) [Đ][e]	$M_{n, NMR}$ (kg mol^{-1})[f]	Wt% PE(v)[g]	DP_{NMR}[h]
**1**-vinyl	94	90	-	52.3	57.8 [1.04]	68.4	21	26-318-26
**1**-COOH	-	-	13	57.0	55.2 [1.12]	73.0	26	-
**2**-vinyl	88	99	-	102	100 [1.05]	100	20	36-472-36
**2**-COOH	n.a.	n.a	12	112	118 [1.20]	108	25	-

[a] Nomenclature: **1**- or **2**-vinyl refers to PE(v)-*b*-PDL-*b*-PE(v) triblock polymers with overall M_n of 60 or 100 kg mol^{-1}, respectively. **1**- or **2**-COOH denotes the corresponding polymers, PE_{COOH}-*b*-PDL-*b*-PE_{COOH}, where the outer block is modified with 3-mercaptopropionic acid (MPA). Reaction conditions: DL ROP: 80 °C, $[DL]_0$ = 2.0 M in toluene/vCHO, 1 h; $[LZnMg(C_6F_5)_2]_0:[BDM]_0:[DL]_0$= 1:4:1000 (**1**-vinyl) or 1:4:2000 (**2**-vinyl); vCHO/PA ROCOP: 100 °C, $[LZnMg(C_6F_5)_2]_0:[BDM]_0:[PA]_0:[vCHO]_0$ = 1:4:200:600 (**1**-vinyl) or 1:4:400:1200 (**2**-vinyl). [b] Determined by ^{1}H NMR spectroscopy ($CDCl_3$) from the relative integrals of resonances at 4.31 and 4.85 ppm for DL and PDL, respectively. [c] Conversion of PA to PE(v) determined from the relative integrals of resonances for PA (8.15 ppm) compared to PE(v) (7.65 ppm) from the ^{1}H NMR spectra ($CDCl_3$). NB. No ether linkages from ROP of vCHO were observed. [d] Based on initial $[cat]_0/[BDM]_0$ ratios and % conv. DL and $[PA+vCHO]_0/[BDM]_0$ ratios and % conv. PA. [e] Estimated by SEC (THF eluent, RI detector *vs* PS standards). [f] Estimated from the relative integration of BDM:vCHO: DL and molar masses of PDL and PE(v) repeat units of 170.3 and 272.3 g mol^{-1}, respectively. [g] Determined from the relative integrals of PE(v) (7.49 ppm) and PDL (4.85 ppm) resonances in the ^{1}H NMR spectra of purified polymer films and using molar masses of PDL and PE(v) repeat units. [h] Degree of polymerization calculated from $M_{n, NMR}$ and wt% PE(v) using repeat unit molar mass (specified above).

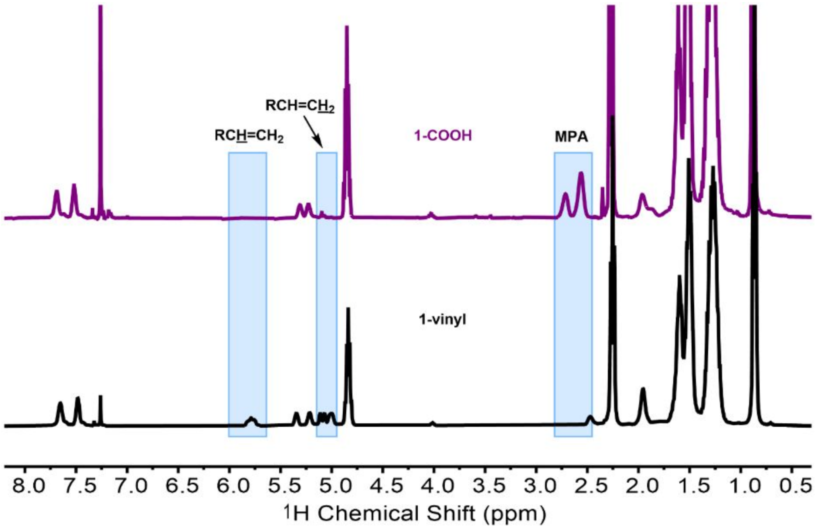

Figure S5. ^{1}H NMR Spectra ($CDCl_3$) comparing PE(v)-PDL-PE(v) (bottom) and PE_{COOH}-PDL-PE_{COOH} (top). Loss of the vCHO vinyl proton at 5.82 ppm (RCH=CH$_2$), alongside the appearance of the substituent propionic acid protons at 2.56 & 2.57 ppm, confirms successful thiol-ene click reaction converting **1**-vinyl to **1**-COOH.

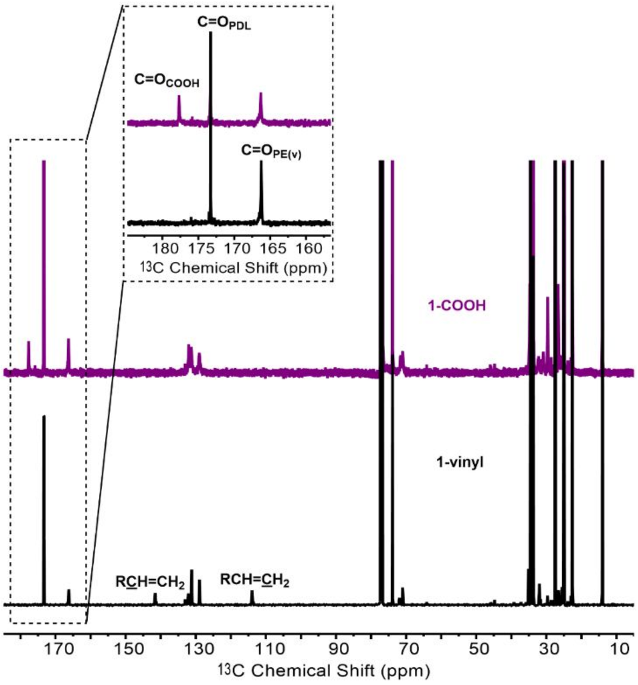

Figure S6. $^{13}C\{^1H\}$ NMR Spectra ($CDCl_3$) of 1-vinyl and 1-COOH. Inset: Carbonyl region showing C=O resonance for PDL at 173.4 ppm and PE (v) 166.4 ppm. For **1-**COOH, the environment at 177.8 ppm is assigned to the carboxylic acid, C=O group. No evidence of transesterification in the carbonyl region is observed in either polymer. Carbon environments at 141.6 ppm (C=<u>*C*</u>HR) and 113.9 ppm (C=<u>*C*</u>H$_2$) in **1**-vinyl are lost on modification with 3-mercaptopropionic acid.

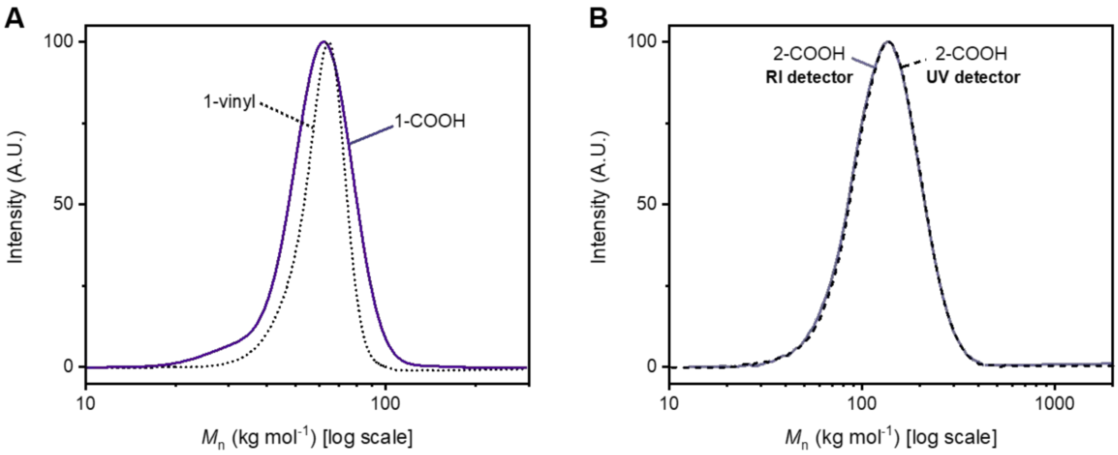

Figure S7. SEC Traces after modification with 3-mercaptopropionic acid (MPA). A: **1**-vinyl series. B: **2**-COOH analyzed by both RI or UV detection (M_n = 118 kg mol^{-1}, $Đ$ 1.20). All traces were measured in THF eluent vs narrow polystyrene standards.

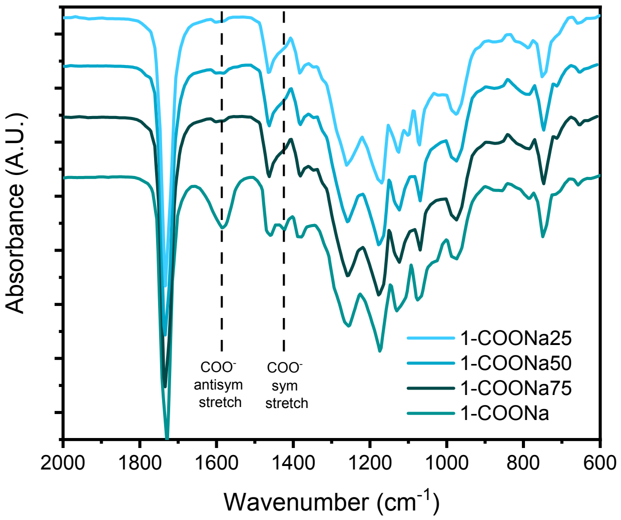

Figure S8. ATR-FTIR Spectra (thin-film) of 1-COOH neutralized with NaOH to different extents. Increasing sodium neutralization from top to bottom: 25, 50, 75 and 100% (**1**-COONa). The intensity ratio of the sodium-carboxylate coordination band at 1580 cm^{-1} and the ester carbonyl stretch at 1730 cm^{-1} increases with sodium content as per the extent of sodium neutralization.

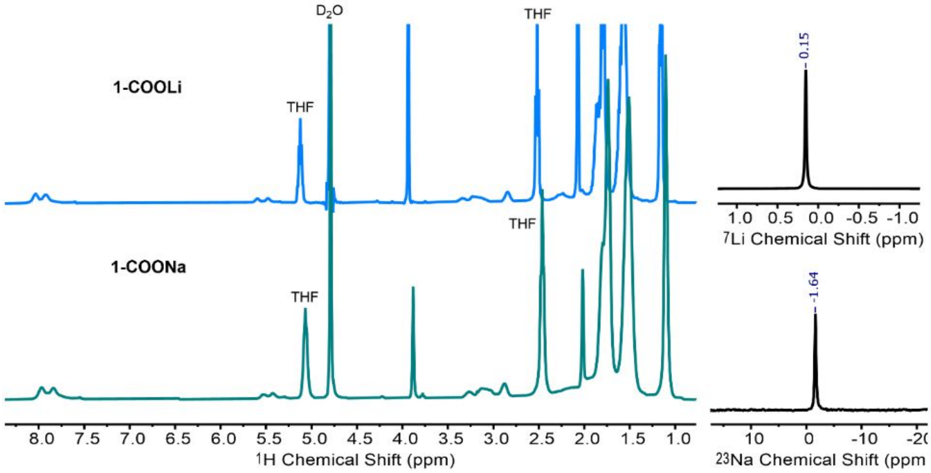

Figure S9. ^{1}H NMR Spectra (THF/D_2O) of 1-COOH after neutralization with LiOH (top) or NaOH (bottom). Right inset: corresponding ^{7}Li and ^{23}Na NMR spectra.

Table S2. ICP Analysis of polymer samples.[a]

Sample	Measured (ppm)	Expected (ppm)
**1**-COOLi	0.389	0.365
**1**-COONa	1.986	1.882
**1**-COONa25	0.037	0.040
**1**-COONa50	0.431	0.399
**1**-COONa75	0.589	0.596
**2**-COONa75	0.899	0.847

[a] Procedure: Polymers (10-120 mg) were dissolved in 2% nitric acid. Na content was corrected against a control sample containing no polymer to account for the Na content in the glass container. Expected values are estimated based on the degree of polymerization of each block, determined by SEC, and assuming 100% carboxylic acid functionalization. Discrepancies between expected and observed Na ppm values are attributed to sodium in the glass vial container, standard errors in weighing small polymer masses and the process of degrading the polymer samples in nitric acid for measurements to be carried out, which may produce imperceptible insoluble products removed by syringe filtration (2 μm pore) before analysis.

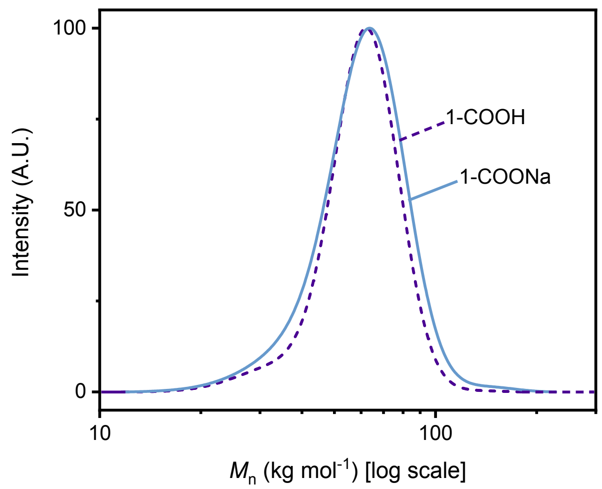

Figure S10. SEC Trace of 1-COONa. The sample was submitted in a water/THF mixture.

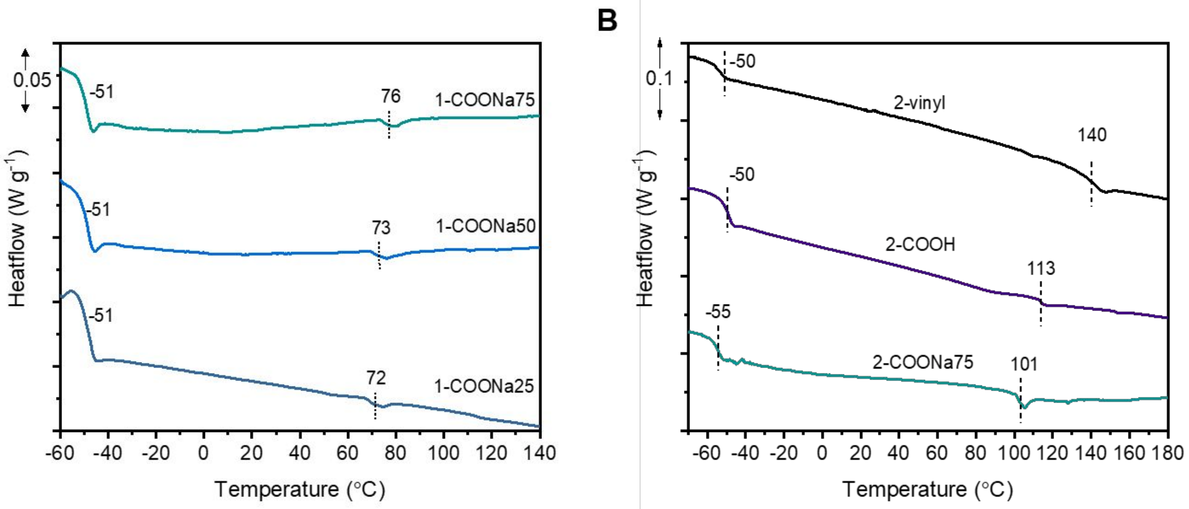

Figure S11. DSC traces of polymer films. Measurements were conducted from -80 to + 200 °C. A: **1**-COOH neutralized to different extents with NaOH. B: Comparison of higher M_n series: **2**-vinyl, **2**-COOH and **2**-COONa75. The reduction in upper T_g on functionalization is attributed to increased degrees of free of the 2-mercapto propanoic acid. On ionization this upper glass transition appears sharper and more defined. The reason for the decrease compared to 2-COOH is unclear but more sensitive DMA measurements show that 2-COONa75 upper T_g is indeed higher than 2-COOH (Figure 5A).

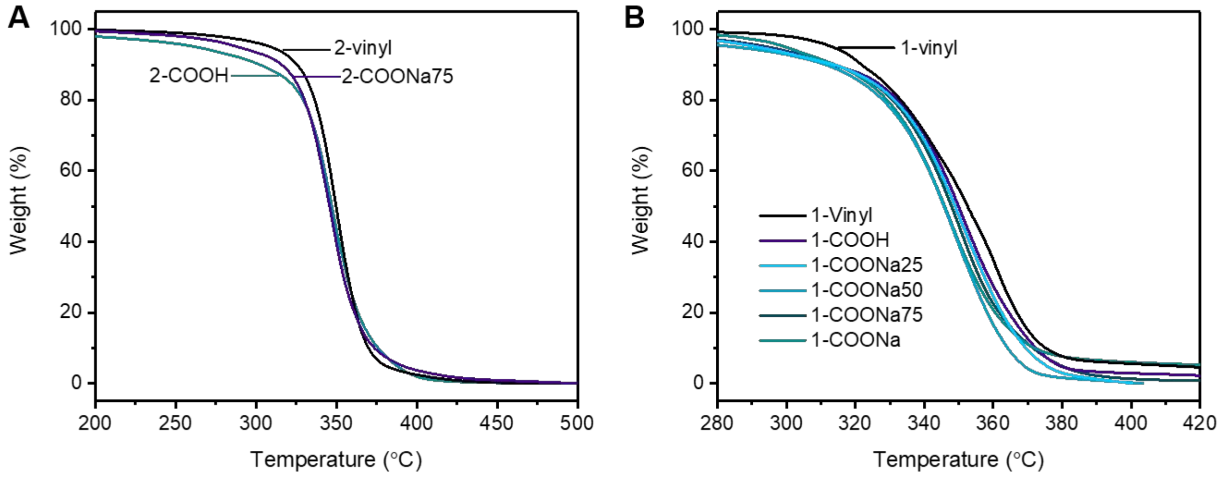

Figure S12. TGA analyses of polymers. A: Comparison of higher M_n series: **2**-vinyl, **2**-COOH and **2**-COONa75. B: **1**-COOH neutralized to different extents with NaOH.

Table S3. Characterization of block copolyester phase morphology by SAXS.

**Polymer**	***d* / nm** [a]	**Observed *q*/*q** ** [b]	**Proposed Morphology**
1-COOH	28.0	1, √3, (√4), (√7)	HEX (weakly ordered)
1-COOLi	26.0	1, (√3), √4, √7, (√9), (√12), √13	HEX
1-COONa	16.3	1, √3, (√4), √7	HEX
2-vinyl	44.6	1, √3	HEX (weakly ordered)
2-COOH	25.8	1, √3	HEX (weakly ordered)
2-COONa75	23.8	1, √3, (√4), √7, (√9), √13	HEX

[a] *d* = domain spacing calculated from the position of the principal scattering peak (*q**). [b] Permitted reflections for the proposed morphology; those missing are in parentheses. HEX = hexagonally packed cylinder morphology.

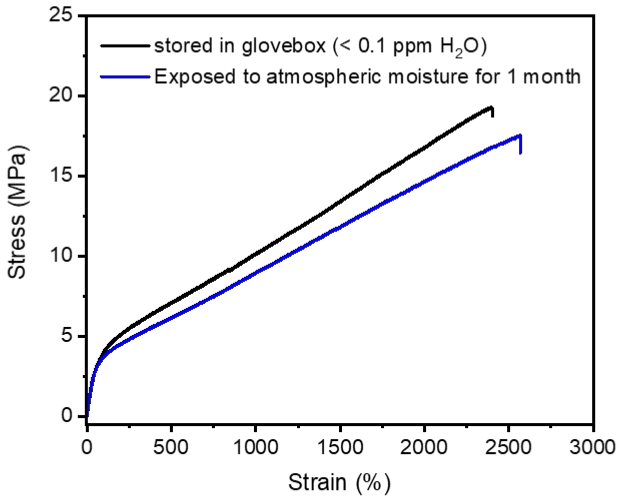

Figure S13. Influence of moisture content on mechanical properties. The stress-strain curve in black is for a sample of **2**-COONa75 stored in a glovebox immediately after drying and before testing. In blue is a sample of **2**-COONa75 stored on the bench for 1 month.

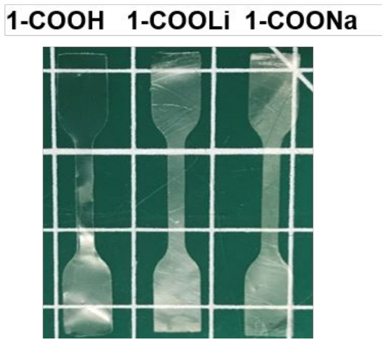

Figure S14. Photo showing the transparency of block polyester TPE ionomers.

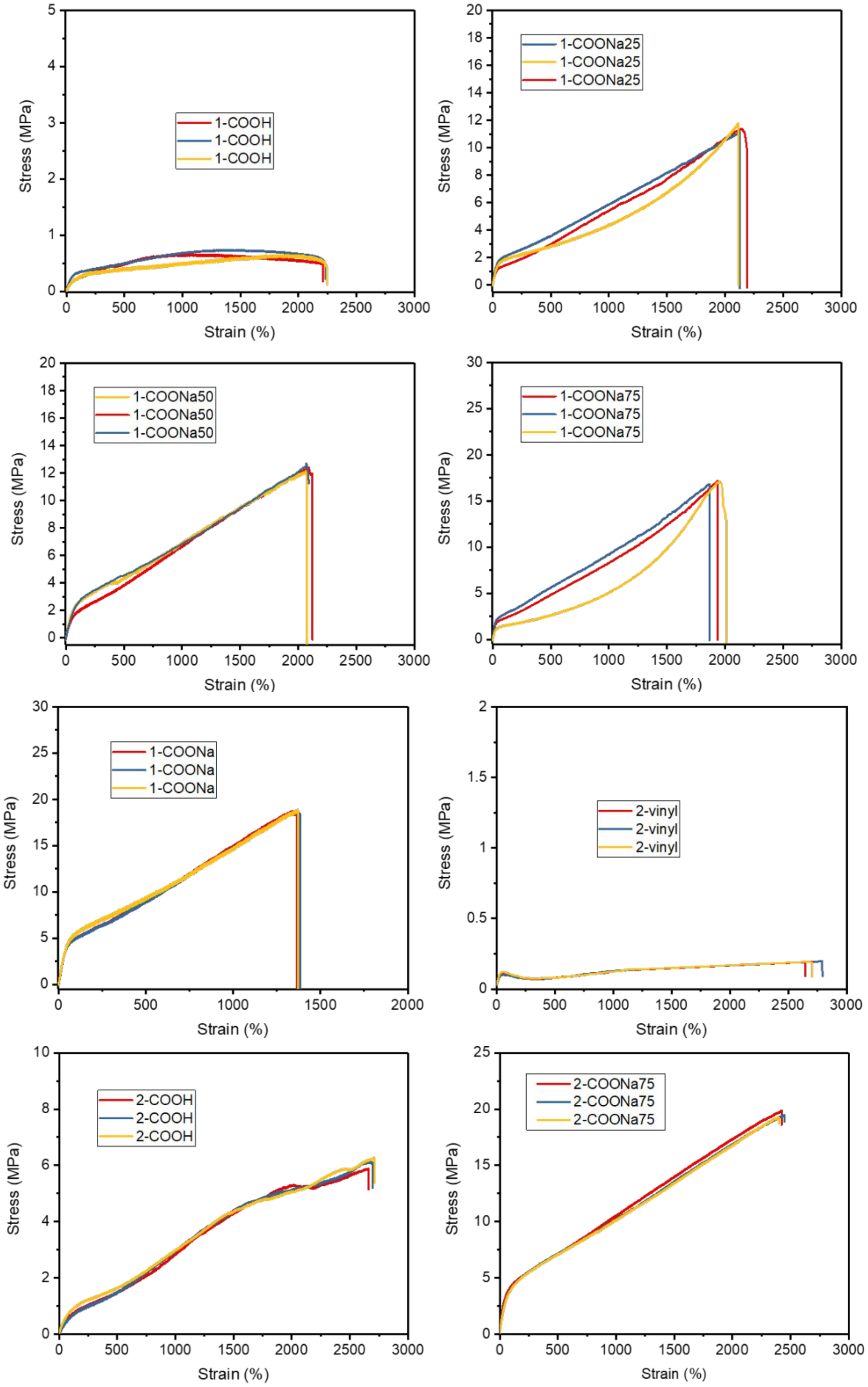

Figure S15. Stress-Strain curves showing repeat measurements. Three repeats are shown for each polymer sample (as labelled).

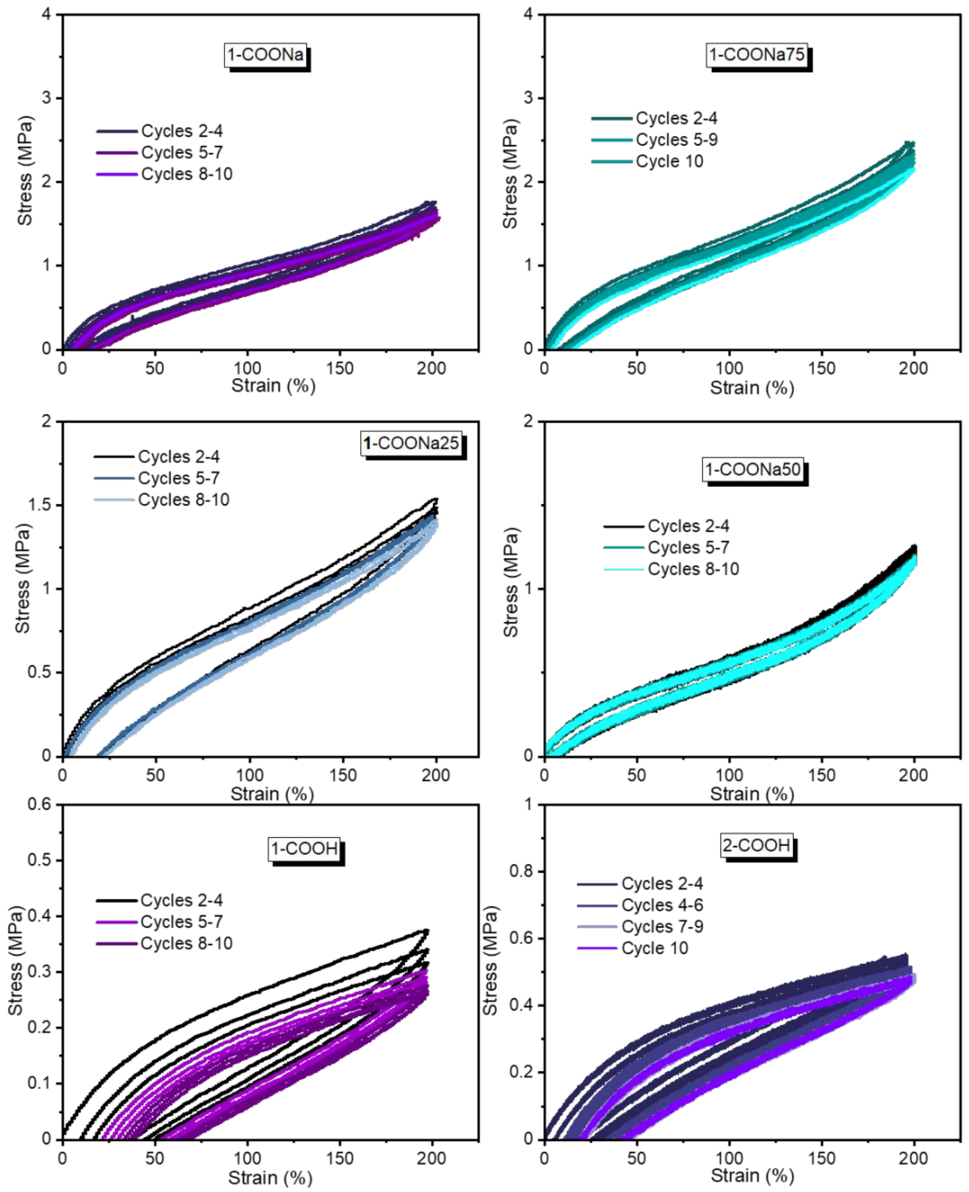

Figure S16. Loading-unloading curves for polymers repeatably stretched to 200% strain. The difference between the first and subsequent loading-unloading cycles is attributed to changes in the polymer microstructure.

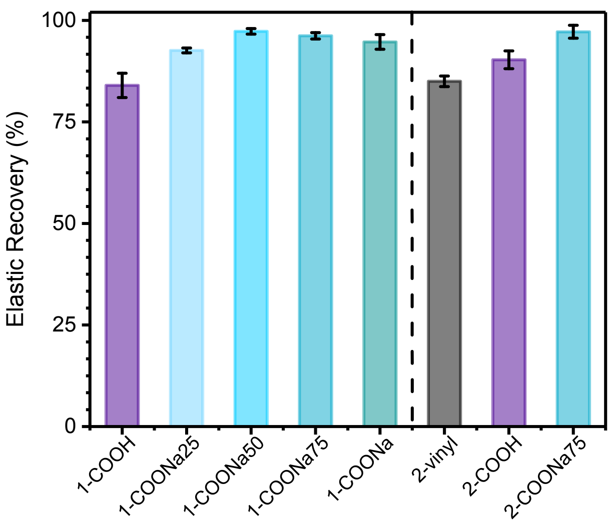

Figure S17. Elastic recovery. Samples were repeatably stretched to 200% strain and allowed to relax. Error bars represent the standard deviation of at least three measurements. The dotted line marks the two polymer series investigated differing in overall M_n.

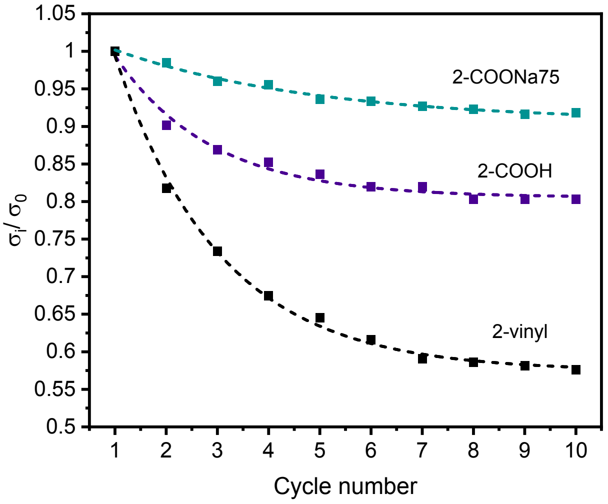

Figure S18. Stress Softening Behaviour during Cyclic Tensile Testing. The maximum stress at 200% strain for each cycle (σ_i) is plotted as a ratio to the original stress at cycle 1 (σ_0).

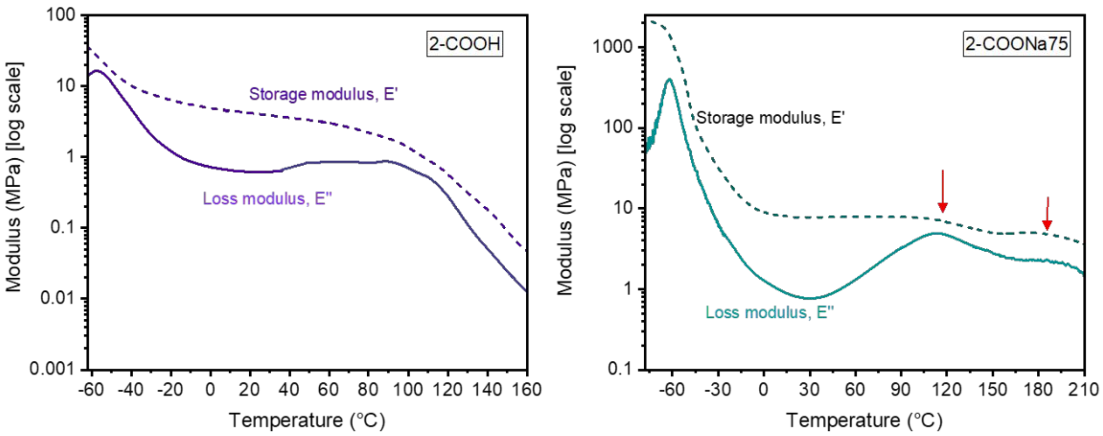

Figure S19. Temperature Dependence of Storage (*E*') and Loss (*E*'') Moduli from DMTA. A rapid drop in *E*' is observed at higher temperatures for **2**-COOH before the sample yields. This behaviour was not observed for **2**-COONa75 and is consistent with sodium interactions between polymer chains.[5-6] The molecular weight between the entanglements (M_e) was estimated ($M_e \sim \rho RT/3E'$) from the plateau in *E*' at T = 298 K using ρ_{PDL} = 0.97 g cm^{-1} and assumes a Poisson ratio, v of 0.5 and no contribution from the rigid microdomains. Using this approach, M_e values were estimated at 1.0 and 2.6 kg mol^{-1} for **2**-COONa75 and **2**-COOH, respectively. Soft block entanglements also contribute to tensile strength (~ 1/M_e) by delaying fracture of the hard domains.[7]

Table S4. Summary of Tensile Mechanical and Thermal data.[a]

Sample	E_y (MPa)	ε_b (%)	σ_b (MPa)	U_T (MJ m^{-3})	T_{g1}, T_{g2} (°C)[b]	$T_{d,5\%}$ (°C)[c]
**1**-COOLi	8.8 ± 2	2217 ± 40	8.7 ± 0.2	105 ± 5	-51, 91	313
**1**-COONa	9.2 ± 1	1363 ± 5	18.5 ± 0.1	153 ± 3	-51, 76	327
**1**-COONa50	6.7 ± 0.1	2089 ± 16	12.4 ± 0.1	130 ± 2	-51, 73	323
**1**-COONa25	3.5 ± 0.2	2138 ± 17	11.3 ± 0.2	121 ± 1	-51, 57	325

[a] Triblock polymers with overall $M_{n,\ SEC}$ of 60 kg mol^{-1} (21 wt% PE(v)) functionalized with lithium or sodium carboxylate. 25 and 50 refer to the extent of carboxylic acid neutralization. Where no number is provided, 100% carboxylic acids are neutralized. E_y = Youngs' modulus, ε_y = elongation at break, σ_b = tensile strength, U_T = tensile toughness measured from the area under the stress-strain curve. [b] DSC. [c] TGA.

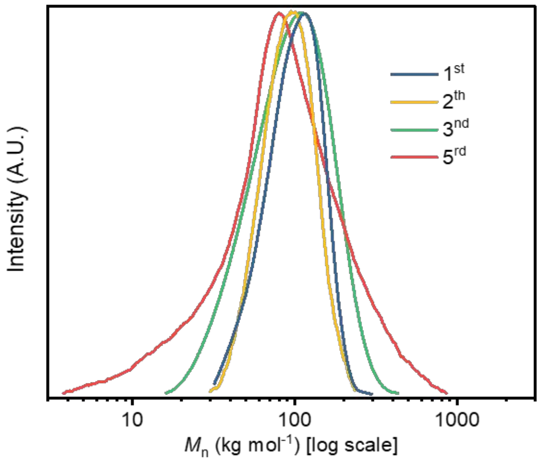

Figure S20. Thermal reprocessing. SEC trace after each reprocessing step. NB. Samples submitted in THF/water mixture.

Table S5: Examples of Ionic TPEs.

Polymer	ε_b (%)	σ_b (MPa)
Zn-SEPDM[8]	691	23
Na-SEPDM[8]	350	6.6
Zn-mEPDM[8]	620	5.8
Zn-XNBR[8]	1150	33
Na-SBR[9]	860	20.8
Li-SBR[9]	730	19.9
Na-SEBS[10]	200-690	13-28
Na-SBS[11]	657-923	12-22
Li-SBS[11]	892	20.2
Suryln®	290-660	18.6-36.5
Iotek®	300-500	26-41

Zn-SEPDM = sulfonated ethylene-propylene-diene rubber (Exxon,TP-301). Zn-mEPDM rubber (Royaltuf-465), Zn-XNBR = Zn-carboxylate crosslinked butadiene rubber (Krynac® 7.5). For SEBS, SBS- sulphonated. 17.2 MPa, 823% with 24 % PS. SBR= styrene butadiene rubber. Suryln® ionomer properties (ethylene-methacrylic acid) from DuPont™ datasheets. Iotek® ionomer properties (ethylene-acrylic acid) from ExxonMobil datasheets.

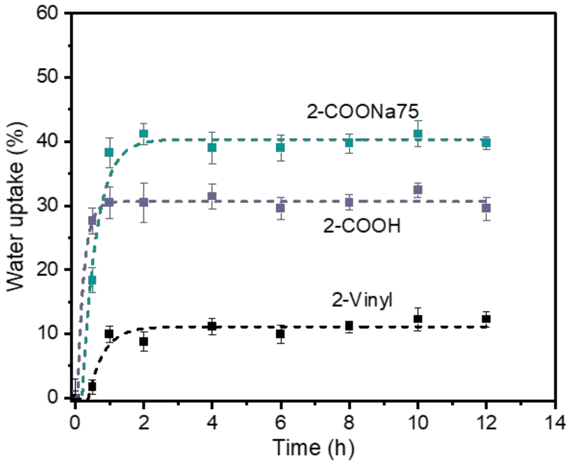

Figure S21. Water Uptake. Polymer films cut into discs (diameter = 16 mm) were submerged in distilled water. Periodically samples were removed, excess surface water was removed with tissue and their wet mass was recorded.

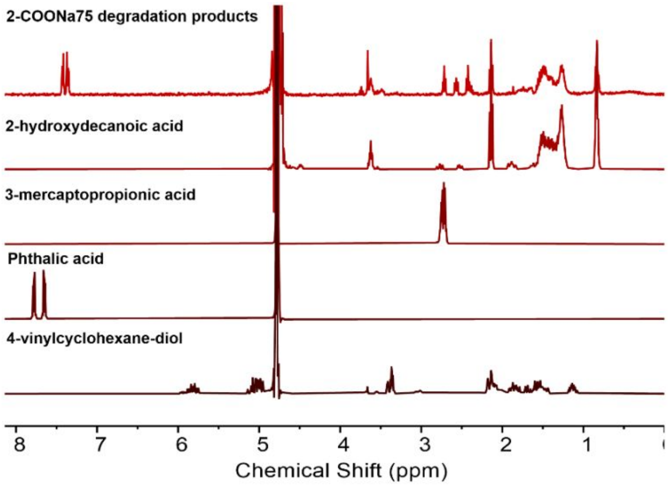

Figure S22. Degradation Products of 2-COONa75 in alkaline media. ^{1}H NMR (D_2O) of residue isolated after the complete mass loss of discs of **2**-COONa75 compared to NMR spectra of pure compounds corresponding to the anticipated degradation products. The shift upfield for the aromatic environments assigned to phthalic acid is attributed to sodium ionization. Re-formation of the free thiol (3-mercaptopropionic acid) is not proposed. The vinyl is fully functionalized in the polymer.

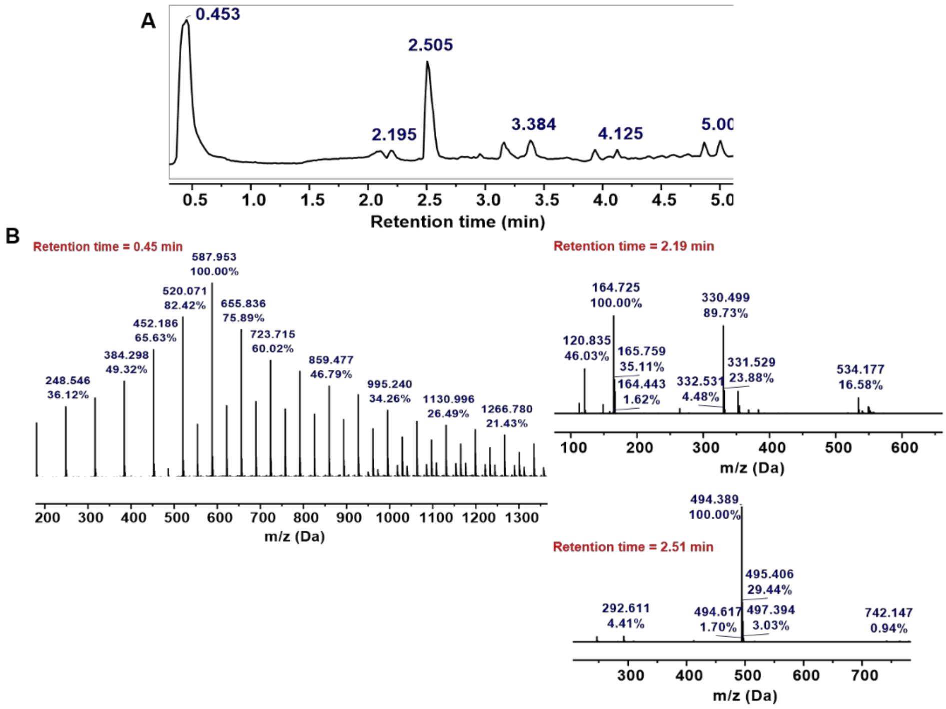

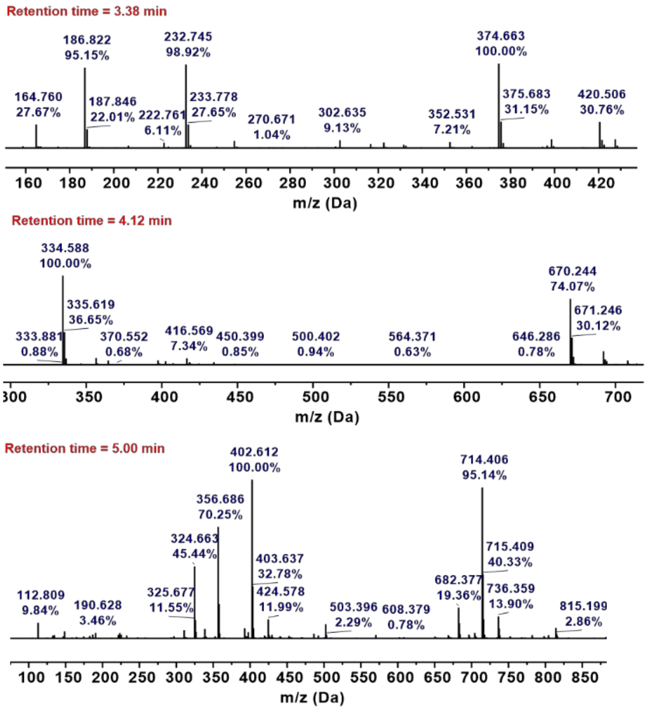

Figure S23. Liquid Chromatography-Mass Spectrometry of degradation products from 2-COONa75. A: Chromatograph of degradation products. B: Mass spectrum corresponding to peaks observed in the chromatogram. The retention time of peaks is provided in red i.e. 0.45 minutes. See Table S6 below for proposed structures.

Table S6: Proposed Assignment of 2-COONa75 Degradation Products in alkaline media.[a]

Proposed Structure	Proposed Species	Expected *m/z*	Observed *m/z* (relative intensity %)	Error (%)	RT (min)
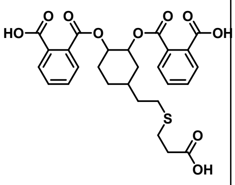	**[M-3H]$^{3-}$**<br>[M-3H+Na+HCOONa]$^{-}$<br>[2M-6H+3Na]$^{3-}$<br>**[M-3H+2Na]$^{-}$**<br>[M-3H+2Na+HCOONa]$^{-}$<br>[M-3H+2Na+2HCOONa]$^{-}$<br>[M-3H+2Na+3HCOONa]$^{-}$	**180.4**<br>316.1<br>383.7<br>**587.1**<br>655.8<br>723.1<br>791.1	**180.7 (27)**<br>316.4 (41)<br>384.3 (49)<br>**587.95 (100)**<br>655.8 (76)<br>723.7 (62)<br>791.6 (55)	-0.17<br>-0.09<br>-0.16<br>-0.14<br>0.00<br>-0.08<br>-0.06	0.45
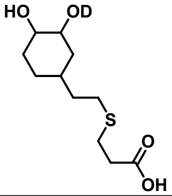	**[M-H]$^{-}$**<br>[M-H+HCOONa]$^{-}$<br>[M-H+2HCOONa]$^{-}$<br>[M-H+3HCOONa]$^{-}$<br>[M-H+4HCOONa]$^{-}$<br>**[M-H+5HCOONa]$^{-}$**<br>[M-H+6HCOONa]$^{-}$	**248.1**<br>316.0<br>384.1<br>452.1<br>520.1<br>**588.0**<br>656.0	**248.5 (36)**<br>316.4 (41)<br>384.3 (49)<br>452.2 (66)<br>520.1 (80)<br>**588.0 (100)**<br>655.8 (76)	-0.16<br>-0.13<br>-0.05<br>-0.02<br>0.00<br>0.00<br>0.03	
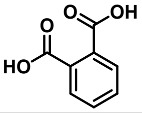	**[M-H]-**<br>[2M-H]$^{-}$	**165.0**<br>331.0	**164.7 (100)**<br>330.5 (89.7)	0.18<br>0.15	2.19
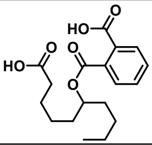	[M-2H+Na+2HCOONa]$^{-}$	493.1	494.4 (100)	-0.26	2.51
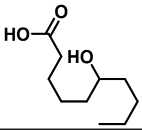	**[M-H]$^{-}$**<br>[2M-3H+Na]$^{-}$<br>[2M-H]$^{-}$	**187.1**<br>231.1<br>375.3	**186.8 (95.2)**<br>232.7 (98.9)<br>374.7 (100)	0.16<br>-0.69<br>0.16	**3.38**

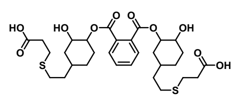	**[M-4H+2Na]$^{2-}$**<br>[M-3H+2Na]$^{-}$	**334.1**<br>670.2	**334.6 (100)**<br>670.2 (74)	-0.15<br>0.00	4.12[b]
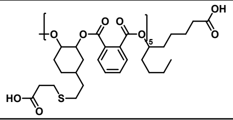	[M-6H+3Na]$^{3-}$	714.2	714.4 (95)	-0.03	5.00[c]
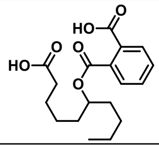	[M-2H+Na]$^{-}$<br>[M-H+HCOONa]$^{-}$	357.1<br>403.1	356.7 (70)<br>402.6 (100)	0.11<br>0.12	

[a] NMR sample (see Figure S22 above) in D_2O diluted in water for analysis by liquid chromatography-mass spectrometry (LC-MS), mode = negative electron-spray ionization, formic acid (HCOONa) used to facilitate ionization.[b] Signal at retention time 3.94 min also corresponds to same m/z values but different relative intensities. [c] Signal at retention time 4.87 min corresponds to the same *m/z*: 402.6 (100), 356.7 (65) and 714.4 (81).

References

1. Sulley, G. S.; Gregory, G. L.; Chen, T. T. D.; Peña Carrodeguas, L.; Trott, G.; Santmarti, A.; Lee, K.-Y.; Terrill, N. J.; Williams, C. K., Switchable Catalysis Improves the Properties of CO2-Derived Polymers: Poly(cyclohexene carbonate-b-ε-decalactone-b-cyclohexene carbonate) Adhesives, Elastomers, and Toughened Plastics. *J. Am. Chem. Soc.* **2020,** *142* (9), 4367-4378.
2. Filik, J.; Ashton, A. W.; Chang, P. C. Y.; Chater, P. A.; Day, S. J.; Drakopoulos, M.; Gerring, M. W.; Hart, M. L.; Magdysyuk, O. V.; Michalik, S.; Smith, A.; Tang, C. C.; Terrill, N. J.; Wharmby, M. T.; Wilhelm, H., Processing two-dimensional X-ray diffraction and small-angle scattering data in DAWN 2. *J. Appl. Crystallogr.* **2017,** *50* (Pt 3), 959-966.
3. Pauw, B. R.; Smith, A. J.; Snow, T.; Terrill, N. J.; Thünemann, A. F., The modular small-angle X-ray scattering data correction sequence. *J. Appl. Crystallogr.* **2017,** *50* (Pt 6), 1800-1811.
4. Spyros, A.; Argyropoulos, D. S.; Marchessault, R. H., A Study of Poly(hydroxyalkanoate)s by Quantitative 31P NMR Spectroscopy: Molecular Weight and Chain Cleavage. *Macromolecules* **1997,** *30* (2), 327-329.
5. Kajita, T.; Tanaka, H.; Noro, A.; Matsushita, Y.; Nozawa, A.; Isobe, K.; Oda, R.; Hashimoto, S., Extremely tough block polymer-based thermoplastic elastomers with strongly associated but dynamically responsive noncovalent cross-links. *Polymer* **2021,** *217*, 123419.
6. Miwa, Y.; Kurachi, J.; Kohbara, Y.; Kutsumizu, S., Dynamic ionic crosslinks enable high strength and ultrastretchability in a single elastomer. *Commun. Chem.* **2018,** *1* (1), 5.
7. Tong, J.-D.; Jerôme, R., Dependence of the Ultimate Tensile Strength of Thermoplastic Elastomers of the Triblock Type on the Molecular Weight between Chain Entanglements of the Central Block. *Macromolecules* **2000,** *33* (5), 1479-1481.
8. Antony, P.; De, S. K., IONIC THERMOPLASTIC ELASTOMERS: A REVIEW. *J. Macromol. Sci. C* **2001,** *41* (1-2), 41-77.
9. Xie, H. Q.; Ao, Z. P.; Guo, J. S., Melt flow and mechanical properties of sulfonated sbr ionomers and their polymer blends. *J. Macromol. Sci. A* **1995,** *34* (3), 249-262.
10. Ghosh, S. K.; De, P. P.; Khastgir, D.; De, S. K., Zinc ionomer based on sulfonated maleated styrene-ethylene/butylene-styrene block copolymer. *Macromol. Rapid Commun.* **1999,** *20* (9), 505-509.
11. Xie, H.-Q.; Liu, D.-G.; Xie, D., Preparation, characterization, and some properties of ionomers from a sulfonated styrene–butadiene–styrene triblock copolymer without gelation. *J. Appl. Polym. Sci.* **2005,** *96* (4), 1398-1404.
